# The cranial osteology of *Tyrannoneustes lythrodectikos* (Crocodylomorpha: Metriorhynchidae) from the Middle Jurassic of Europe

**DOI:** 10.7717/peerj.608

**Published:** 2014-10-02

**Authors:** Davide Foffa, Mark T. Young

**Affiliations:** 1 School of GeoSciences, Grant Institute, University of Edinburgh, Edinburgh, United Kingdom; 2 School of Earth Sciences, University of Bristol, Bristol, United Kingdom; 3 School of Ocean and Earth Sciences, National Oceanography Centre, University of Southampton, Southampton, United Kingdom

**Keywords:** Skull, Middle Jurassic, Marcrophagy, Metriorhynchidae, *Tyrannoneustes*

## Abstract

*Tyrannoneustes lythrodectikos* is one of numerous metriorhynchid crocodylomorph species known from the Oxford Clay Formation of England (Callovian-Oxfordian; Middle-Late Jurassic). This taxon is of evolutionary importance, as it is the oldest and most basal known macrophagous metriorhynchid. It has a mosaic of plesiomorphic and derived feeding related characteristics, including: teeth with microscopic, poorly formed and non-contiguous denticles; increased tooth apicobasal length; ventrally displaced dentary tooth row (increased gape); reduced dentary tooth count; and a proportionally long mandibular symphysis. However the type specimen, and current referred specimens, all lack a preserved cranium. As such, the craniofacial morphology of this taxon, and its potential feeding ecology, remains poorly understood. Here we describe two skulls and two lower jaws which we refer to *T. lythrodectikos*. Previously these specimens were referred to ‘*Metriorhynchus*’ *brachyrhynchus*. They share with the *T. lythrodectikos* holotype: the in-line reception pits on the dentary, dorsal margin of the surangular is strongly concave in lateral view, and the most of the angular ventral margin is strongly convex. Based on our description of these specimens, the skull of *T. lythrodectikos* has three autapomorphies: very long posterior processes of the premaxilla terminating in line with the 4th or 5th maxillary alveoli, deep lateral notches on the lateral surface of the maxillary with reception pits for dentary teeth, and the premaxilla forms the anterior margin of the first maxillary alveoli. Our description of the cranial anatomy of *Tyrannoneustes lythrodectikos* confirms that some macrophagous characteristics evolved during the Middle Jurassic, and were not exclusive to the clade Geosaurini. Moreover, the skulls further highlight the mosaic nature of *Tyrannoneustes lythrodectikos* and wide-gape macrophagous evolution in Geosaurinae.

## Introduction

Metriorhynchid crocodylomorphs were a highly aberrant, but very successful clade. They diversified and radiated extensively during the Middle and Late Jurassic, and continued to diversify throughout the Early Cretaceous (e.g., Eudes-Deslongchamps, 1867–1869; Fraas, 1902; Andrews, 1913; Hua et al., 2000; Gasparini & Iturralde-Vinent, 2001; Buchy et al., 2007; Buchy, 2008; Lepage et al., 2008; Pol & Gasparini, 2009; Young et al., 2010; Fernández et al., 2011; Parrilla-Bel et al., 2013; Herrera, Gasparini & Fernández, 2013; Young et al., 2014). During their evolution, metriorhynchids evolved numerous osteological and soft-tissue adaptations which enabled them to successfully exploit the marine realm, these included: hypocercal tail with a soft-tissue upper lobe, hydrofoil-like forelimbs, osteoporotic-like lightening of the skeleton, neomorphic openings for salt excretion with large nasal salt-glands that were most likely highly vascularised, and poorly developed olfactory bulbs (e.g., Fraas, 1902; Arthaber, 1906; Andrews, 1913; Hua & Buffrenil, 1996; Fernández & Gasparini, 2000; Fernández & Gasparini, 2008; Fernández & Herrera, 2009; Young et al., 2010; Leardi, Pol & Fernández, 2012; Herrera, Fernández & Gasparini, 2013a).

The taxonomy of the metriorhynchids from the Oxford Clay Formation and contemporaneous deposits of northern France is confusing, particularly in regards to the ‘brevirostrine’ species. These short/broad-snouted specimens have usually been referred to the species ‘*Metriorhynchus*’ *brachyrhynchus* (Adams-Tresman, 1987; Young et al., 2013a). However, not all of the specimens with cranial material can easily be referred to ‘*M*.’ *brachyrhynchus*. Two such specimens are NHMUK PV R3939 and PETMG:R176. The former preserves an incomplete cranium in two separate pieces (the rostrum and the dorsoventrally flattened orbitotemporal area), an incomplete mandible, four cervical vertebrae and four dorsal vertebrae (all eight of which are distorted). The latter specimen is an almost complete skull dorsoventrally distorted in the postorbital area and only missing few elements from the braincase, the posterior palatal surface and left temporal area. Alongside these two crania we describe PETMG:R60, a complete but distorted lower jaw from King’s Dyke (near Peterborough), which have been associated to PETMG:R176. This association is anonymous, as in an old PETMG register there is a note: “may fit R176”. We agree that they are likely from the same individual, based upon their comparable size and sharing *Tyrannoneustes lythrodectikos* autapomorphies. However, due to the lack of detailed historical information on these specimens, we cannot be certain they are from the same individual. Finally, we also refer a complete left mandibular ramus to *Tyrannoneustes lythrodectikos* (CAMSM J64267).

NHMUK PV R3939 was purchased by the then British Museum (Natural History) from Mr Alfred Leeds in December 1911. It was first mentioned by Andrews (1913; 199), and he considered NHMUK PV R3939 to be: “probably of this species” (‘*Metriorhynchus*’ *brachyrhynchus*). The second skull (PETMG:R176) comes from an unspecified location and has never been properly described. JB Delair (1960, unpublished manuscript mentioned by Cross in the PETMG catalogue) described it as: “most of a large skull, as preserved 2′ 7″ long with some broken teeth”, while T Cross (1975, unpublished: 16) attributed it to the Phillips Collection of Peterborough Museum. Both Adams-Tresman (1987) and Vignaud (1995) considered PETMG:R176 to belong to ‘*Metriorhynchus*’ *brachyrhynchus*, although the principal components analysis of Vignaud (1995) showed that PETMG:R176 did not group with the other ‘brevirostrine’ specimens, especially when comparing axes 1–3 and 2–3. Young et al. (2010) listed PETMG:R176 as a referred specimen of *Suchodus brachyrhynchus*. The isolated lower jaw (CAMSM J64267) also has never been described, and was listed under *Metriorhynchus*/*Suchodus brachyrhynchus* by Wilkinson, Young & Benton (2008) and Young et al. (2010). Here we disagree, and instead demonstrate that these specimens pertain to *Tyrannoneustes lythrodectikos* based upon cranial and mandibular autapomorphies.

Based on comparisons with PETMG:R176, a private collection specimen previously referred to *Metriorhynchus superciliosus* (Lepage et al., 2008: 71–73) from Basse-Normandie France, is here referred to *Tyrannoneustes lythrodectikos* based on tooth count, alveolar size, inter-alveolar spacing, maxillary-premaxillary suture morphology and the shape of the supraorbital notch. The skull also lacks an apomorphy of Metriorhynchinae: two non-midline anterior palatine processes. This skull is not included herein as it resides in a private collection in France. However, it was extensively figured by Lepage et al. (2008).

PETMG:R176 and NHMUK PV R3939 are the first *T. lythrodectikos* specimens that preserve the cranium. Our description reveals a number of characteristics unique to this species, which allow us to differentiate *T. lythrodectikos* from the contemporaneous and more commonly discovered species ‘*M*’. *brachyrhynchus*. Moreover, our description of these specimens helps elucidate the evolutionary timings of adaptations to wide-gape macrophagy in geosaurine metriorhynchids.

## Systematic Palaeontology

**Table:** 

Superorder Crocodylomorpha Hay, 1930 (*sensu*Walker, 1970)
Infraorder Thalattosuchia Fraas, 1901 (*sensu*Young & Andrade, 2009)
Family Metriorhynchidae Fitzinger, 1843 (*sensu*Young & Andrade, 2009)
Subfamily Geosaurinae Lydekker, 1889 (*sensu*Young & Andrade, 2009)


*Tyrannoneustes* Young et al., 2013a

**ZooBank Life Science Identifier (LSID) for genus** urn:lsid:zoobank.org:act:E0B2711D-6B35-4062-ACF2-FF975DCEE06C

**Type species.** *Tyrannoneustes lythrodectikos*Young et al., 2013a

**Geological range.** Middle-upper Callovian (possibly to upper Oxfordian).

**Geographical range.** Europe (England and France). An isolated tooth from Poland has been referred to this genus (Young et al., 2013a).

**Diagnosis.** Same as the only known species.

*Tyrannoneustes lythrodectikos* Young et al., 2013a

**Table:** 

v	*1913*	*Metriorhynchus brachyrhynchus* (Eudes-Deslongchamps) – Andrews, p. 199. (*partim*)
v	*1987*	*Metriorhynchus brachyrhynchus* (Eudes-Deslongchamps) – Adams-Tresman, p. 182, 184. (*partim*)
v	*2008*	*Metriorhynchus superciliosus* (Blainville) – Lepage et al., p. 71–73, Figs. 1–6.
v	*2008*	*Metriorhynchus brachyrhynchus* (Eudes-Deslongchamps) – Wilkinson, Young & Benton, p. 1331. (*partim*)
v	*2010*	*Suchodus brachyrhynchus* (Eudes-Deslongchamps) – Young et al., p. 855. (*partim*)
v	*2012a*	*Tyrannoneustes* – Young et al., p. 1155. (*nomen nudum*)
v	*2012b*	‘Mr Leeds’ specimen’ – Young et al., p. 2.
v*	*2013a*	*Tyrannoneustes lythrodectikos* gen. et sp. nov. – Young et al., p. 478, Figs. 3–18.


**ZooBank Life Science Identifier (LSID) for species**

urn:lsid:zoobank.org:act:B6036E1E-9EFE-4A25-B934-EA8800002E6C

**Holotype.** GLAHM V972 – right mandibular ramus (angular, dentary, splenial, surangular, eight teeth), fragment of jugal bar (R), quadratojugal (R, fragment), vertebrae: cervical (4), dorsal (9), caudal (20), numerous dorsal vertebrae transverse processes fragments; ribs: axis (R), middle cervical (1R, 2L), anterior dorsal (proximal half of 1R, 1L), middle dorsal (10L, 6L); ilium (R), ischium (L); numerous broken, unplaced fragments.

**Holotype locality.** Fletton, Cambridgeshire, England, United Kingdom.

**Holotype horizon.** Peterborough Member, Oxford Clay Formation (Cox, Hudson & Martill, 1992).

**Referred specimens**. From the Peterborough Member of the Oxford Clay Formation (Peterborough, Cambridgeshire, United Kingdom; middle Callovian): CAMSM J64267–a complete left mandibular ramus; GLAHM V1145–numerous isolated teeth, left humerus, coracoids, femur, ilium; GLAHM V1399/9–isolated tooth; GLAHM V1402/5–isolated tooth; GLAHM V1430–isolated tooth; GLAHM V1436–numerous isolated teeth; NHMUK PV R3939–incomplete skull, mandible, with eight distorted vertebrae (four of which are cervical vertebrae and the other four are thoracic vertebrae), and isolated fragments (including parts of cervical ribs); PETMG:R176–almost complete skull and PETMG:R60–an associated complete, but distorted mandible.

**Figure 1:**
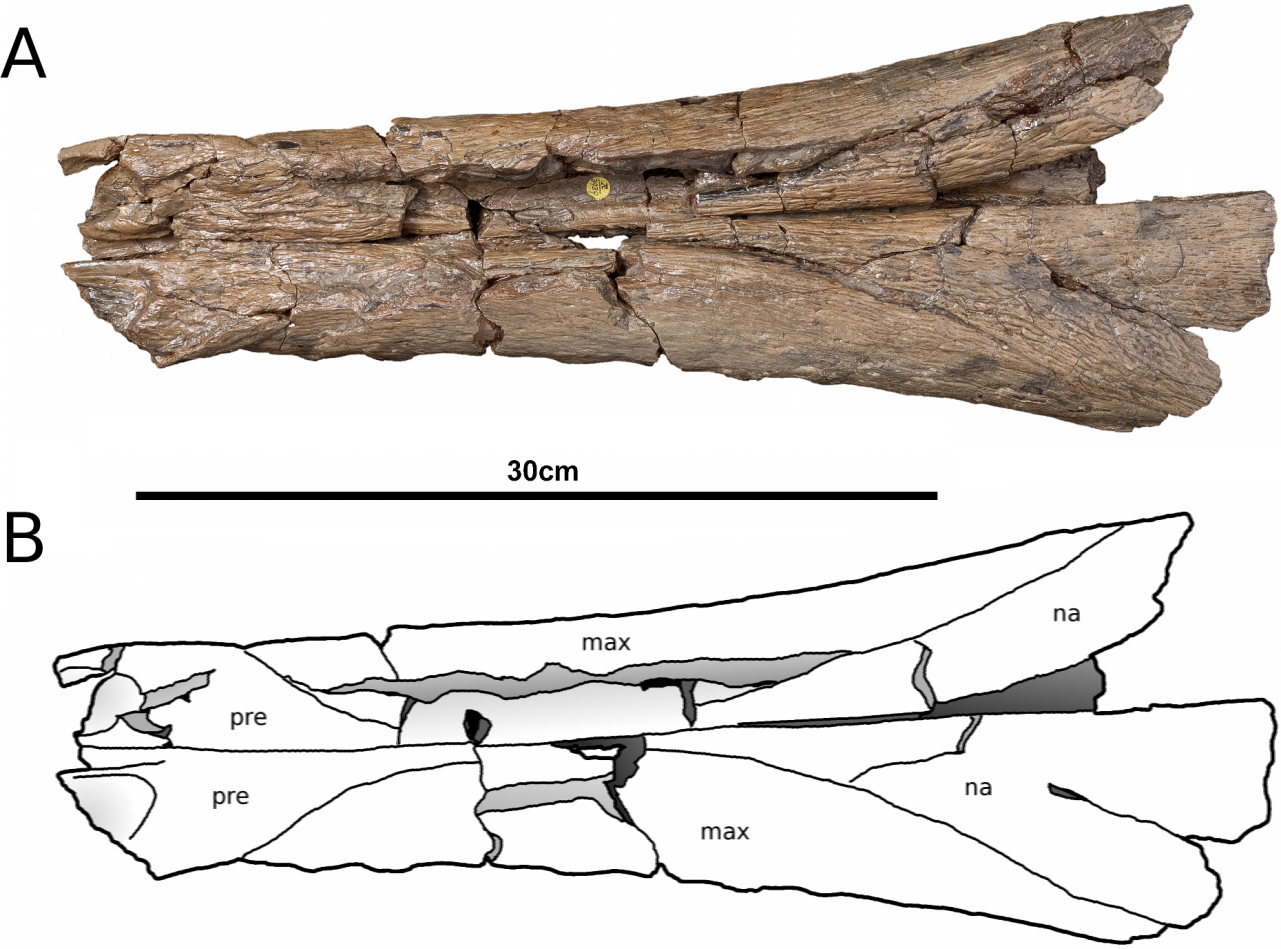
*Tyrannoneustes lythrodectikos*, NHMUK PV R3939. Skull, rostrum. (A) Dorsal view photograph; (B) dorsal view line drawing. Refer to the main text for the abbreviations list.

**Figure 2:**
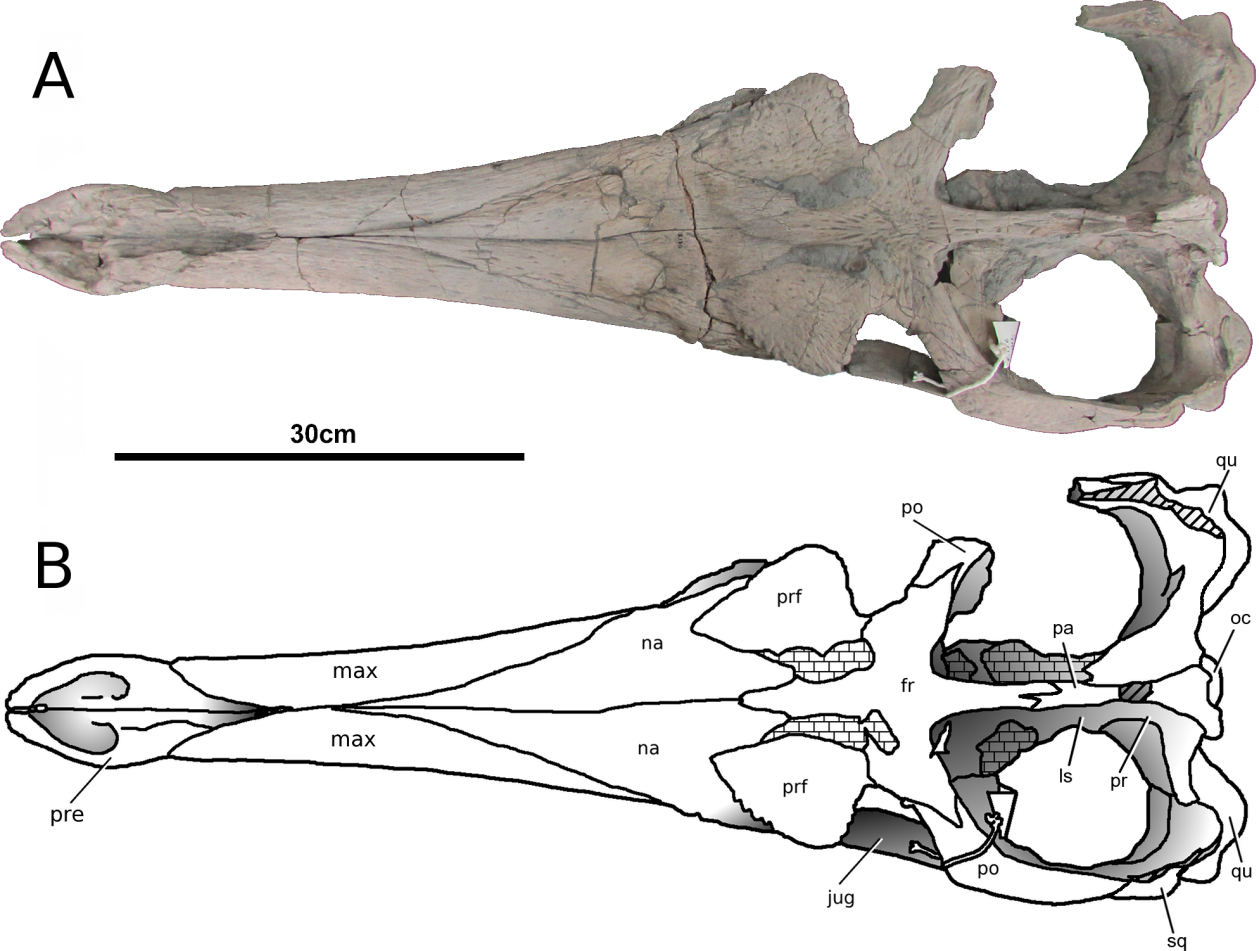
*Tyrannoneustes lythrodectikos*, PETMG:R176. Skull. (A) Dorsal view photograph; (B) dorsal view line drawing. Refer to the main text for the abbreviations list.

**Figure 3:**
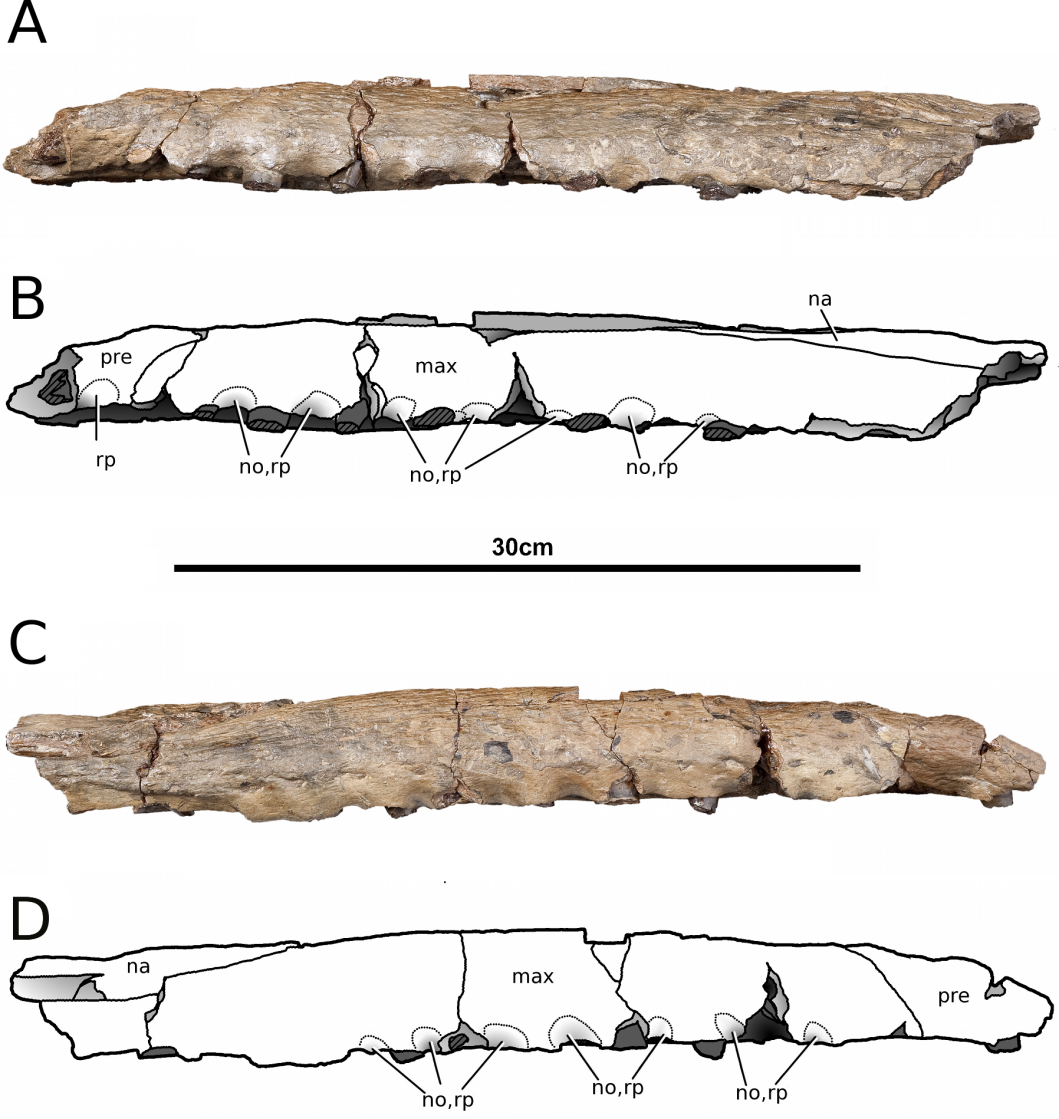
*Tyrannoneustes lythrodectikos*, NHMUK PV R3939. Skull, rostrum. (A) Left lateral view photograph; (B) left lateral view line drawing; (C) right lateral view photograph; (D) right lateral view line drawing. Refer to the main text for the abbreviations list.

**Figure 4:**
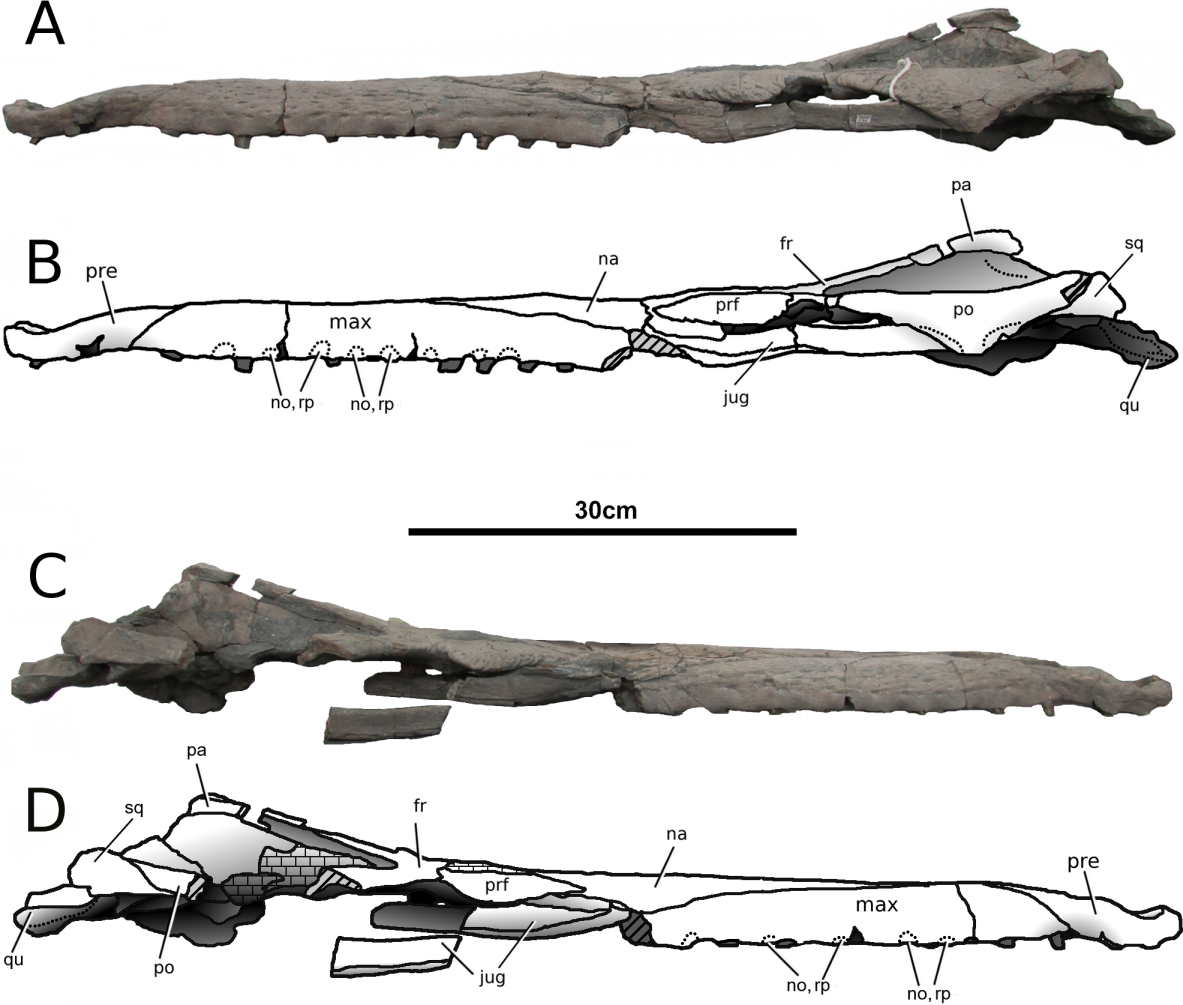
*Tyrannoneustes lythrodectikos*, PETMG:R176. Skull. (A) Left lateral view photograph; (B) left lateral view line drawing; (C) right lateral view photograph; (D) right lateral view line drawing. Refer to the main text for the abbreviations list.

**Figure 5:**
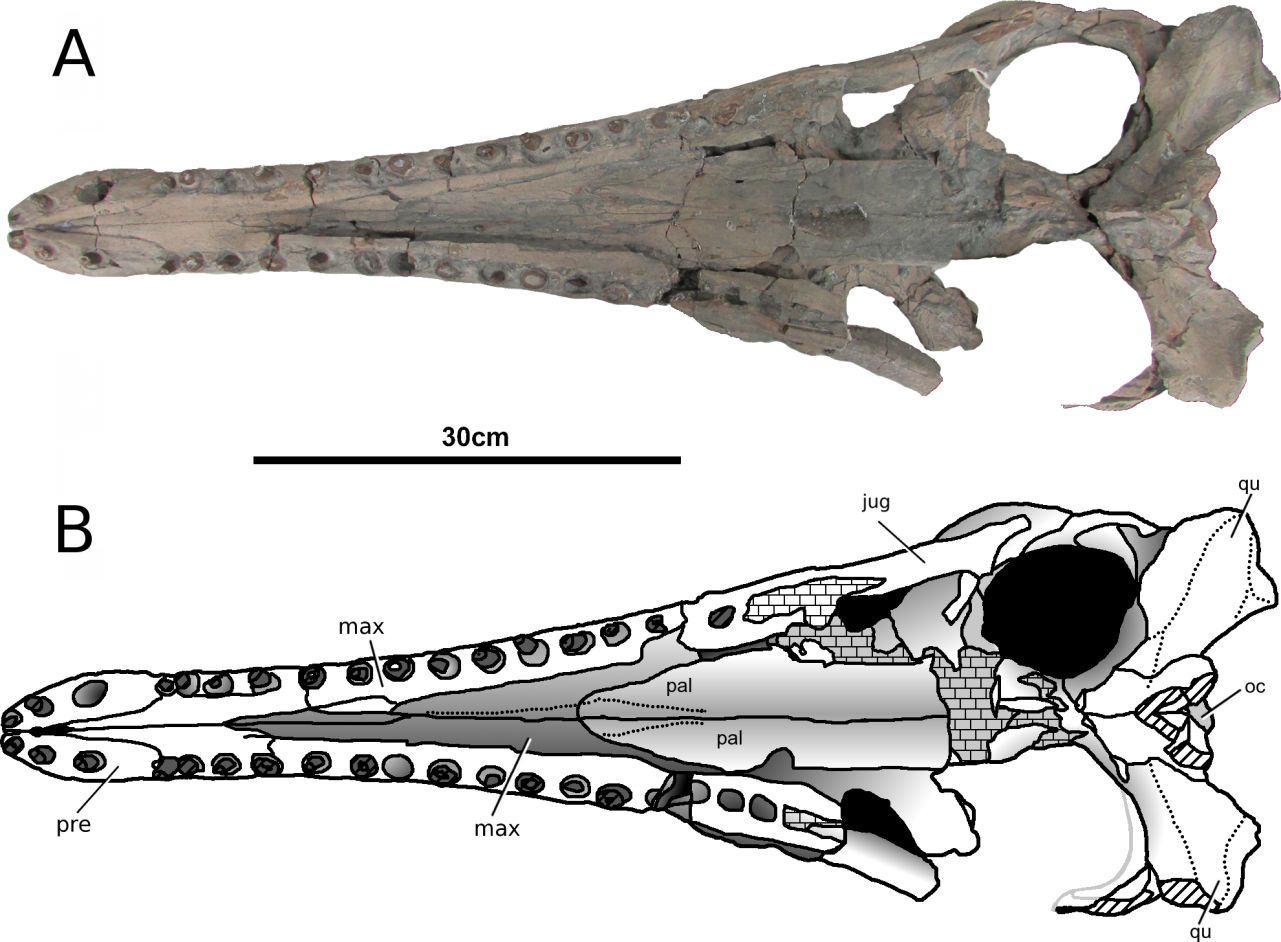
*Tyrannoneustes lythrodectikos*, PETMG:R176. Skull. (A) Ventral view photograph; (B) ventral view line drawing. Refer to the main text for the abbreviations list.

**Figure 6:**
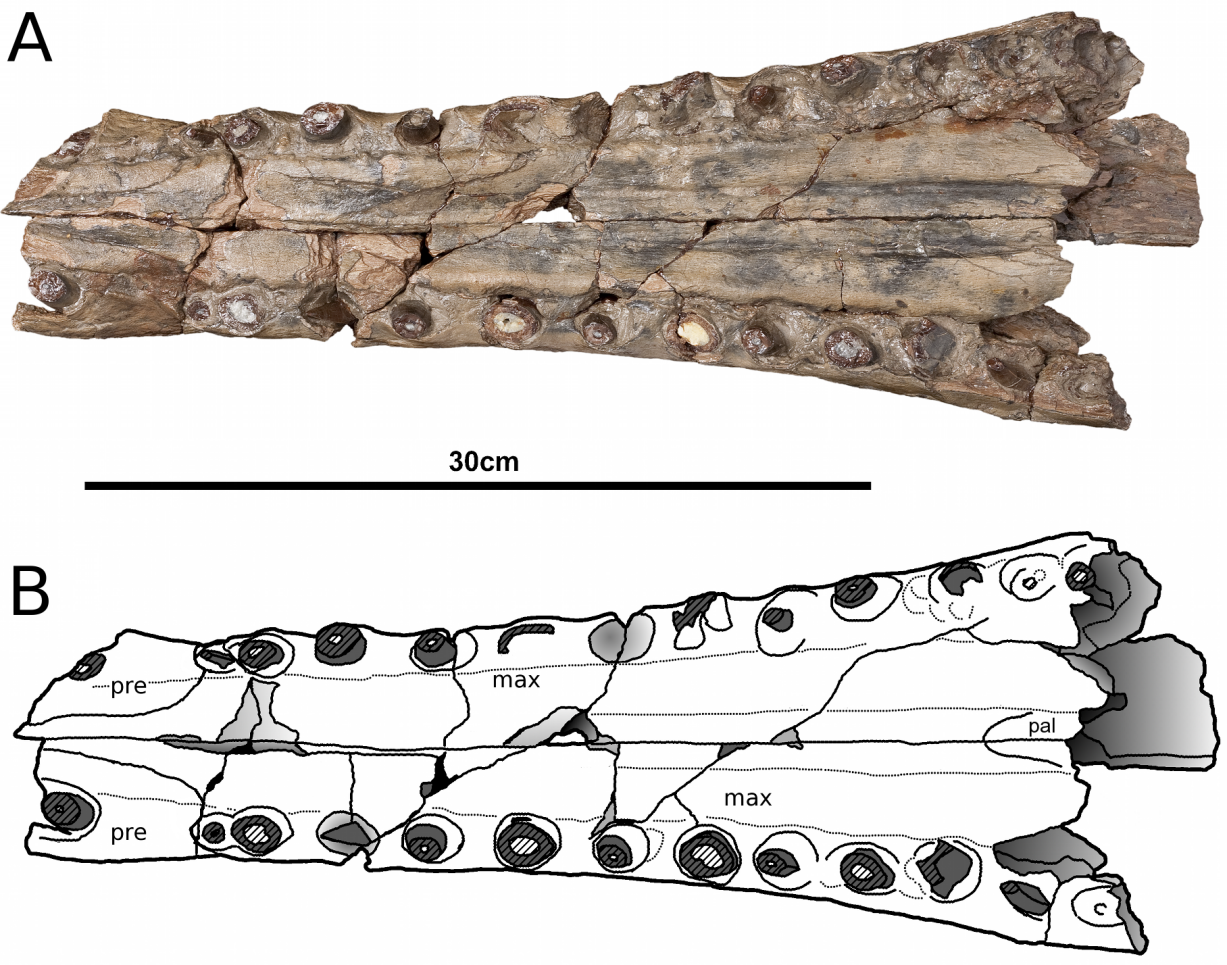
*Tyrannoneustes lythrodectikos*, NHMUK PV R3939. Skull, rostrum. (A) Ventral view photograph; (B) ventral view line drawing. Refer to the main text for the abbreviations list.

**Figure 7:**
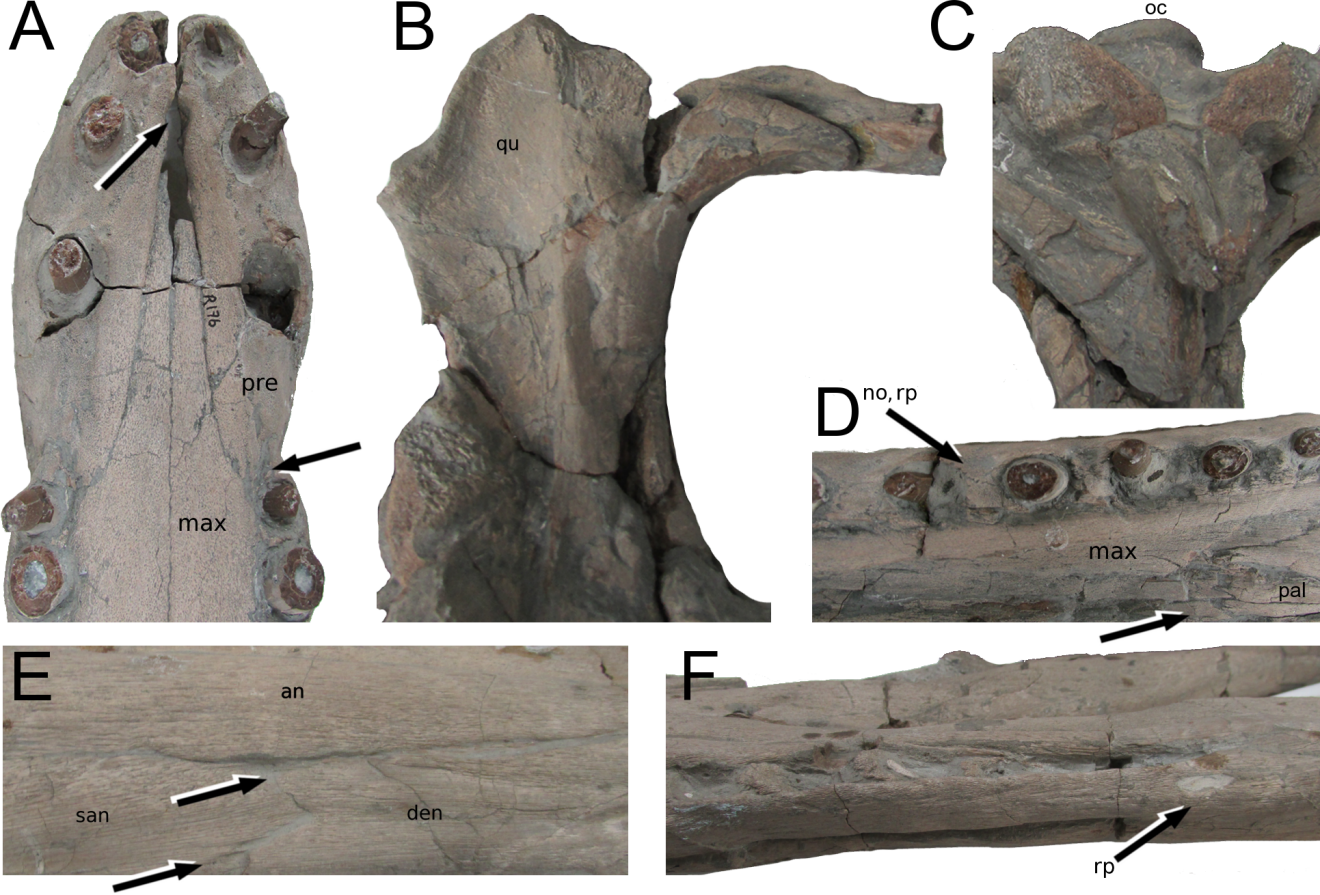
*Tyrannoneustes lythrodectikos*, PETMG:R176 and PETMG:R60. Specific details of skull and mandible. (A)–(D), PETMG:R176; (E)–(F), PETMG:R60. (A) skull, ventral view: incisive foramen (in between the second premaxillary alveoli) and the premaxillary-maxillary suture partial participation to the first maxillary alveolus (M1); (B) left quadrate ventral view; (C) occipital area, ventral view; (D) skull, ventral view: reception pits, deep notches and palatal-maxillary suture; (E) surangular-angular suture; (F) reception pits in the posterior tooth row of the left mandibular ramus. Refer to the main text for the abbreviations list.

**Figure 8:**
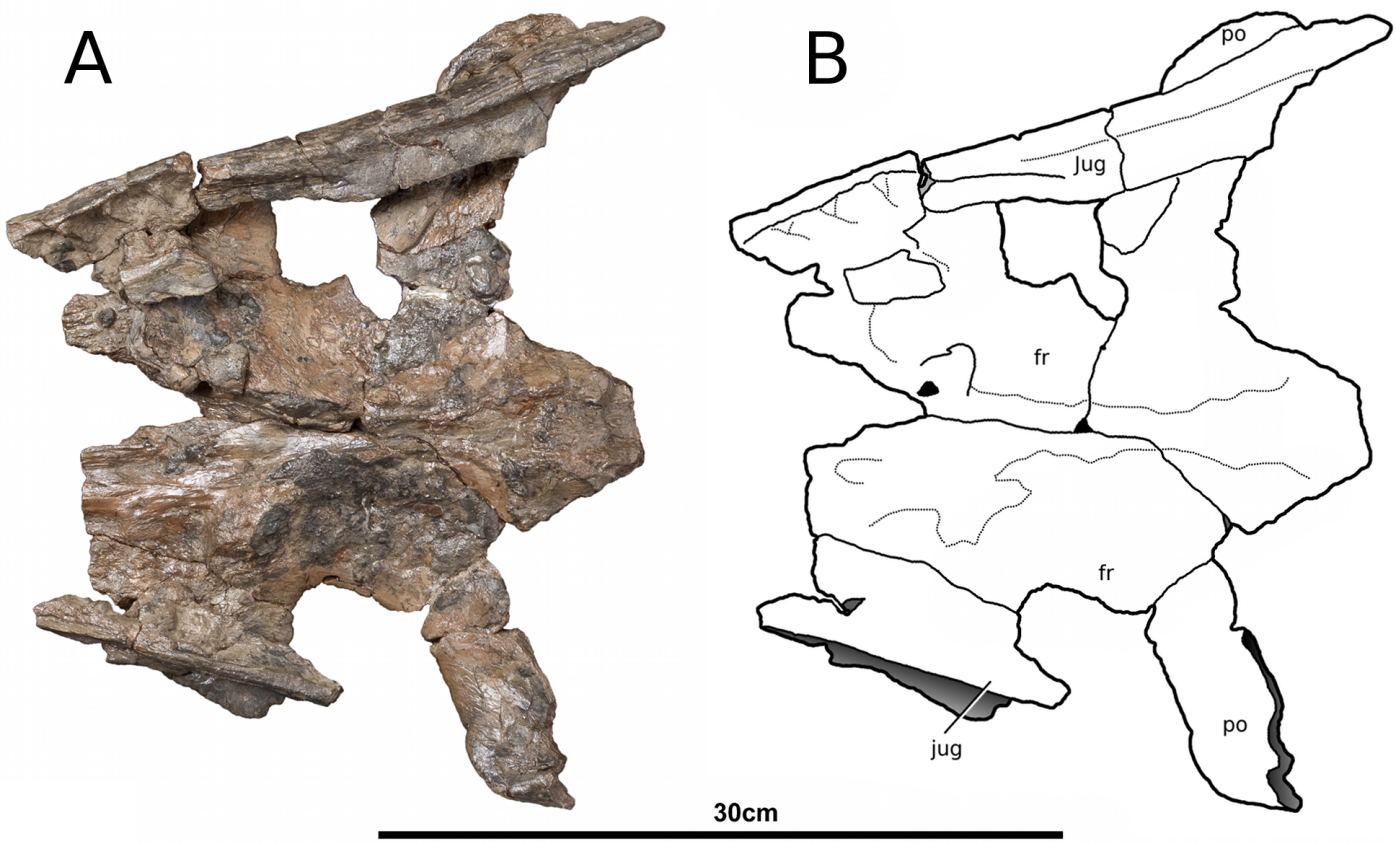
*Tyrannoneustes lythrodectikos*, NHMUK PV R3939. Skull, orbital area. (A) Ventral view photograph; (B) ventral view line drawing. Refer to the main text for the abbreviations list.

**Figure 9:**
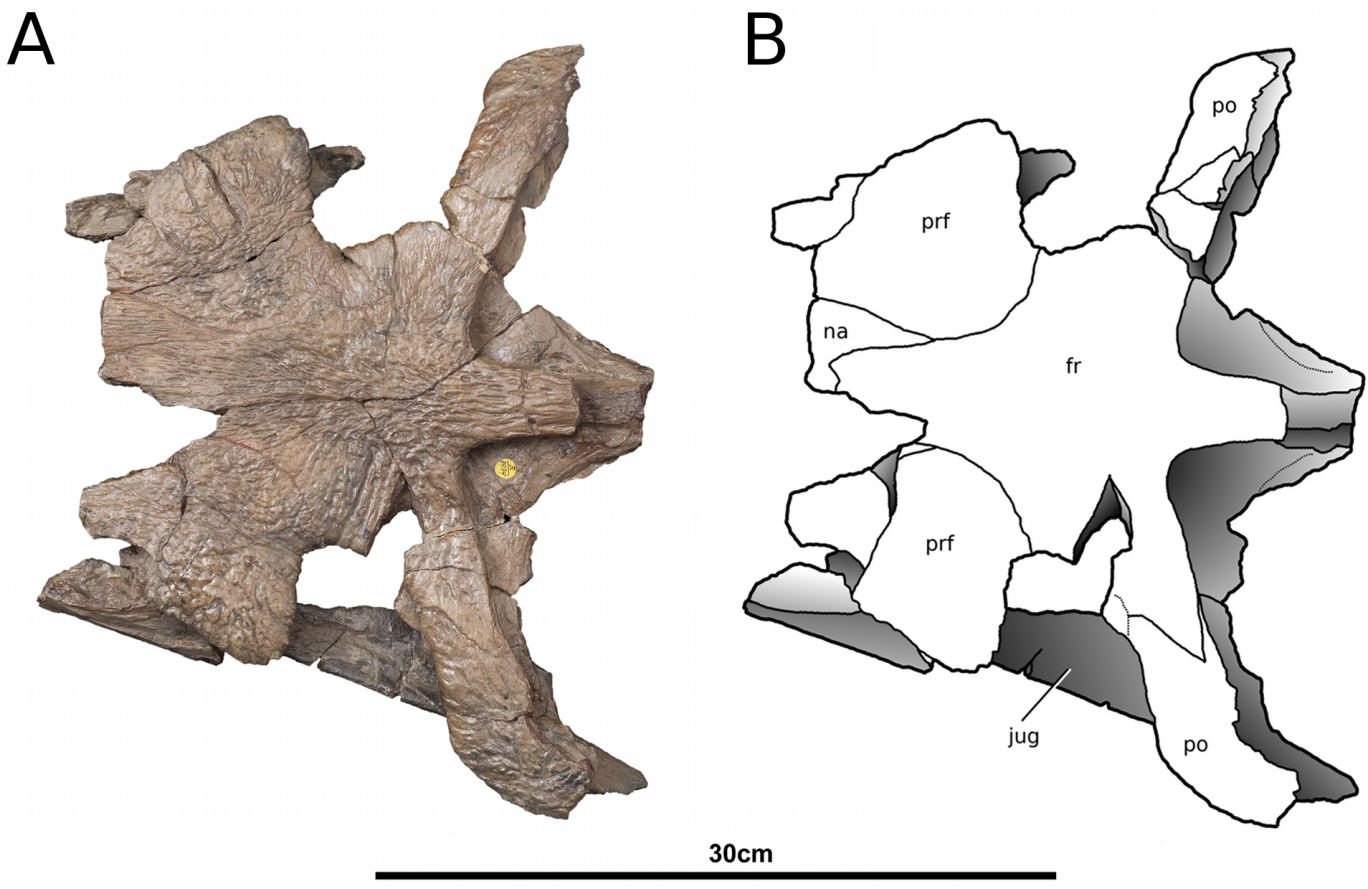
*Tyrannoneustes lythrodectikos*, NHMUK PV R3939. Skull, orbital area. Dorsal view photograph; (B) dorsal view line drawing. Refer to the main text for the abbreviations list.

**Figure 10:**
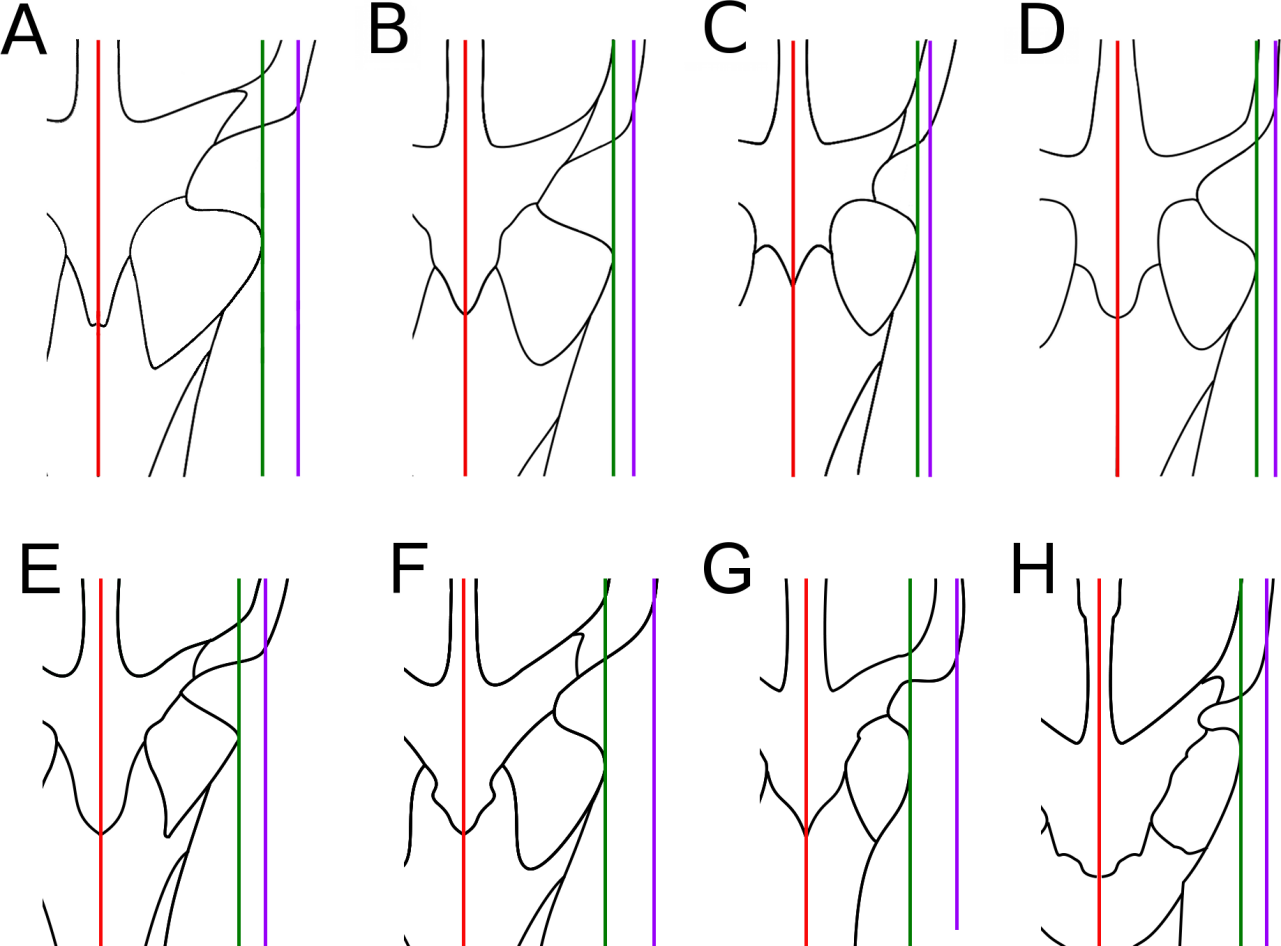
Comparative metriorhynchid skull orbitotemporal area, dorsal view. (A) *Tyrannoneustes lythrodectikos* NHMUK PV R3939; (B) ‘*Metriorhynchus*’ *brachyrhynchus* NHMUK PV R3700; (C) *Gracilineustes leedsi* NHMUK PV R3540; (D) *Metriorhynchus superciliosus* NHMUK PV R2054; (E) *Torvoneustes coryphaeus* SEC K1863; (F) *Torvoneustes carpenteri* BRSMG Ce17365; (G) *Geosaurus grandis* BSPG AS-VI-1; (H) *Dakosaurus andiniensis* MOZ 6146P. Red, sagittal midline plane; green, most lateral sagittal plane on the prefrontal; purple, sagittal plane through maximum curvature of the supratemporal fenestra.

**Figure 11:**
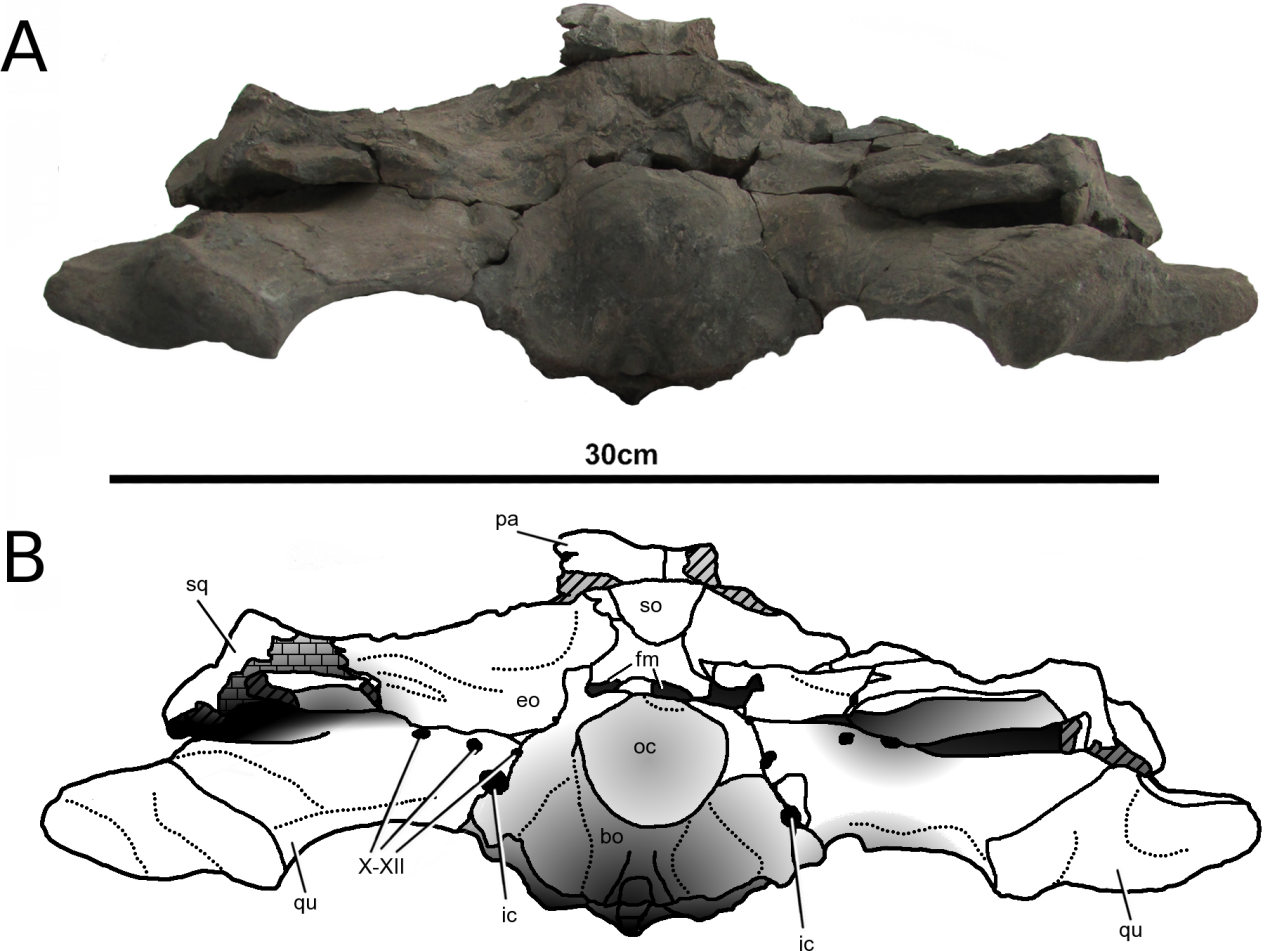
*Tyrannoneustes lythrodectikos*, PETMG:R176. Skull. (A) Occipital view photograph; (B) occipital view line drawing. Refer to the main text for the abbreviations list.

**Figure 12:**
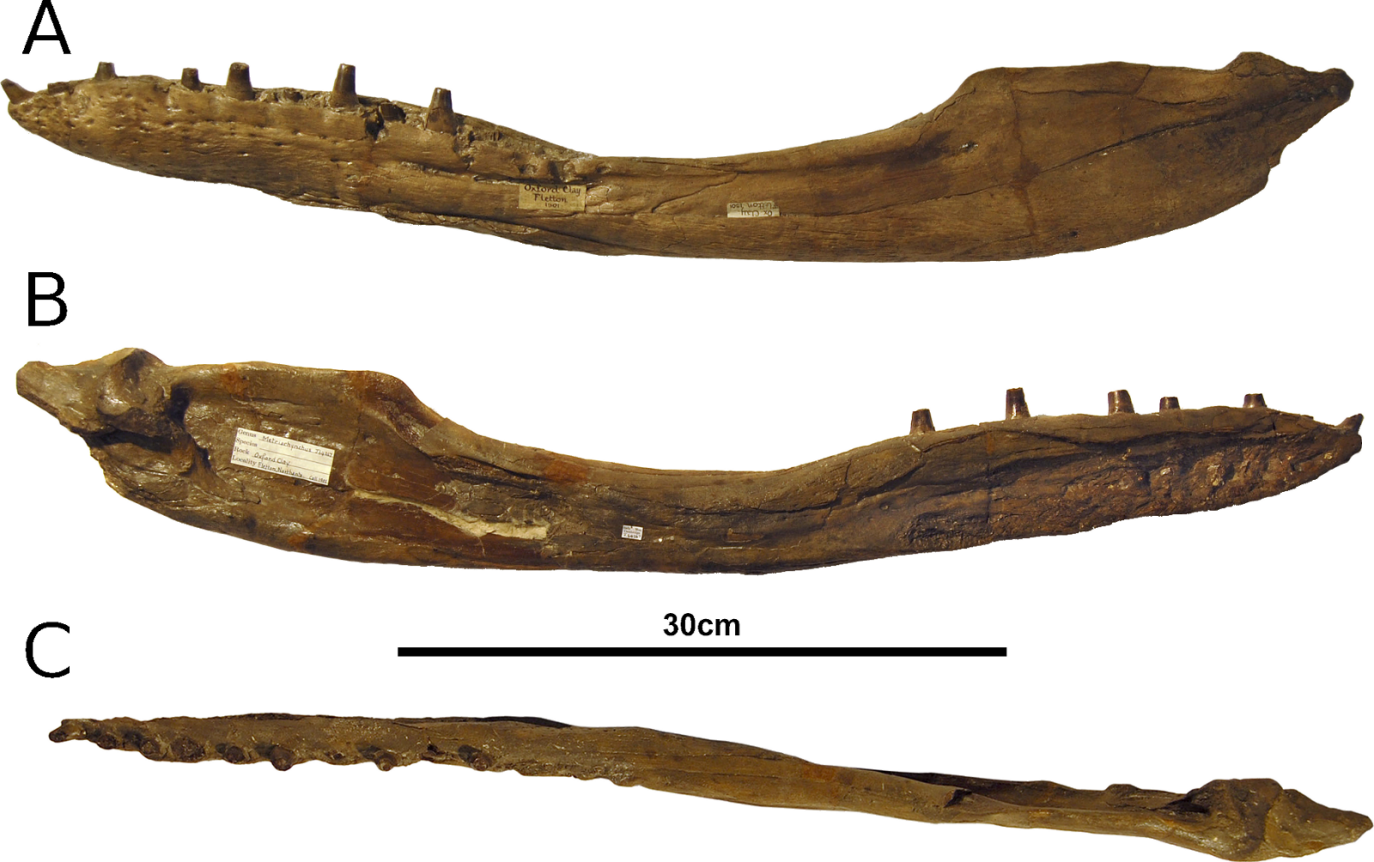
*Tyrannoneustes lythrodectikos*, CAMSM J64267. Mandible, left ramus. (A) Lateral view photograph; (B) medial view photograph; (C) dorsal view photograph.

**Figure 13:**
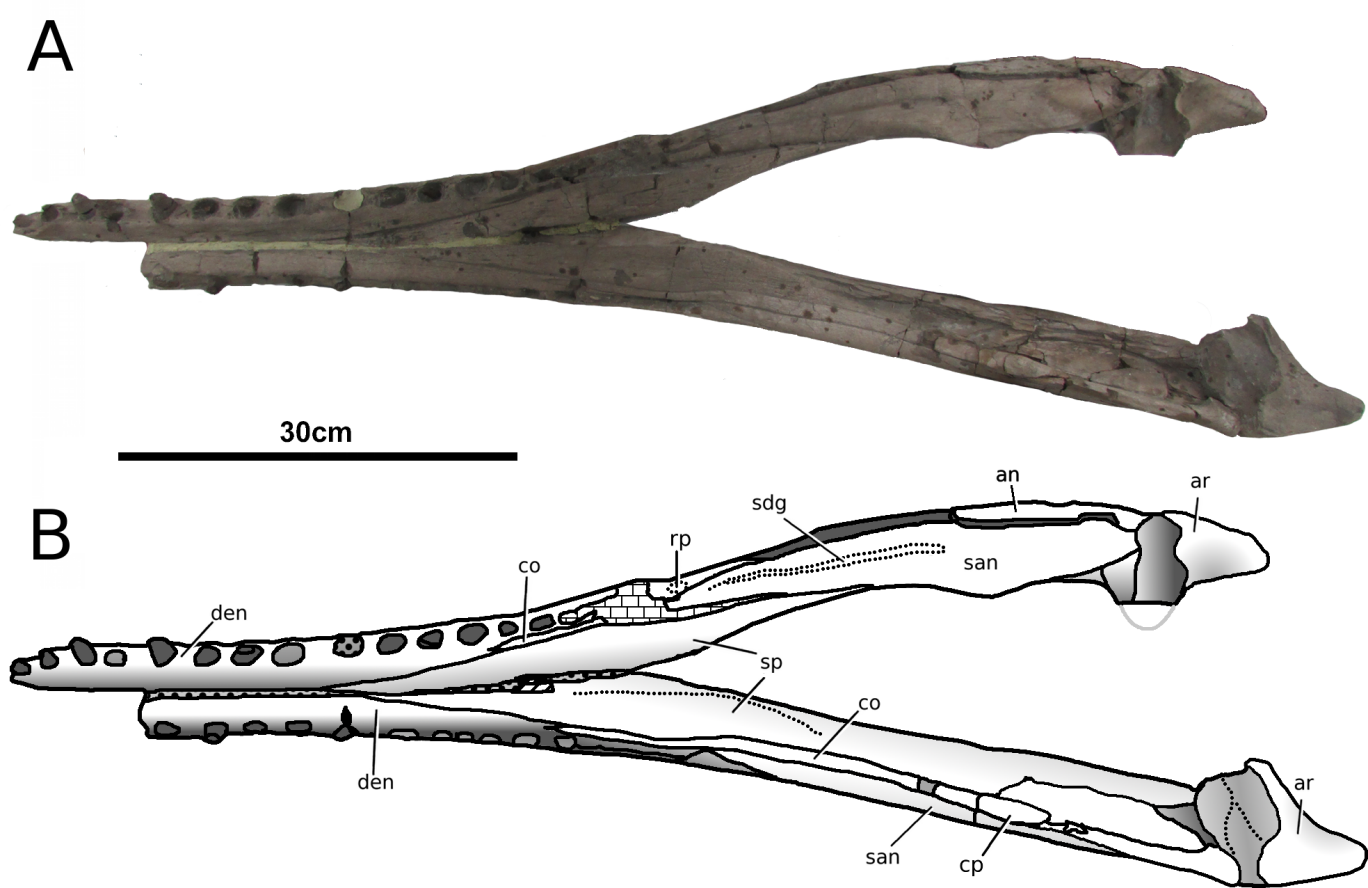
*Tyrannoneustes lythrodectikos*, PETMG:R60. Mandible. (A) Dorsal view photograph; (B) dorsal view line drawing. Refer to the main text for the abbreviations list.

**Figure 14:**
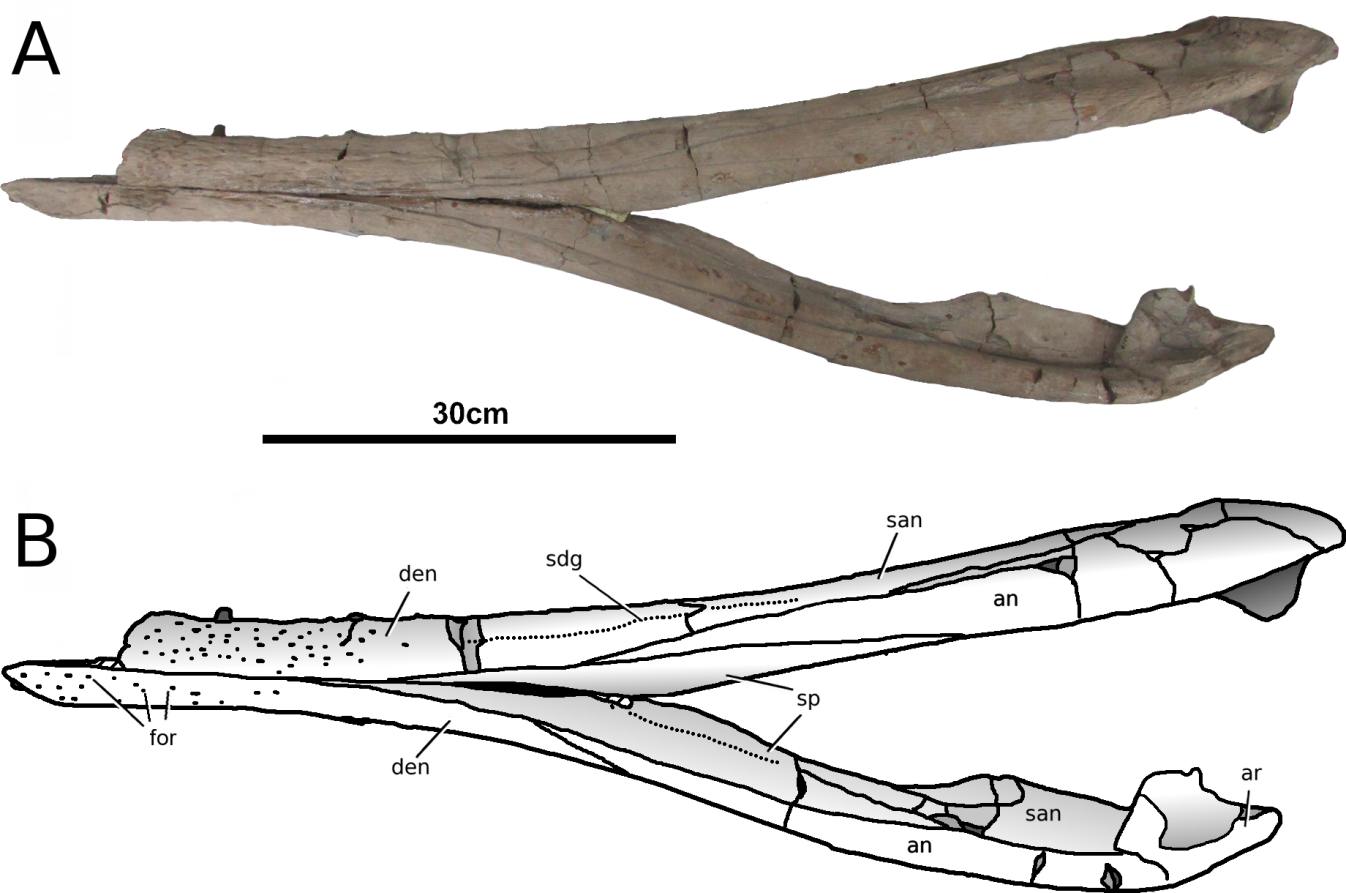
*Tyrannoneustes lythrodectikos*, PETMG:R60. Mandible. (A) Ventral view photograph; (B) ventral view line drawing. Refer to the main text for the abbreviations list.

**Figure 15:**
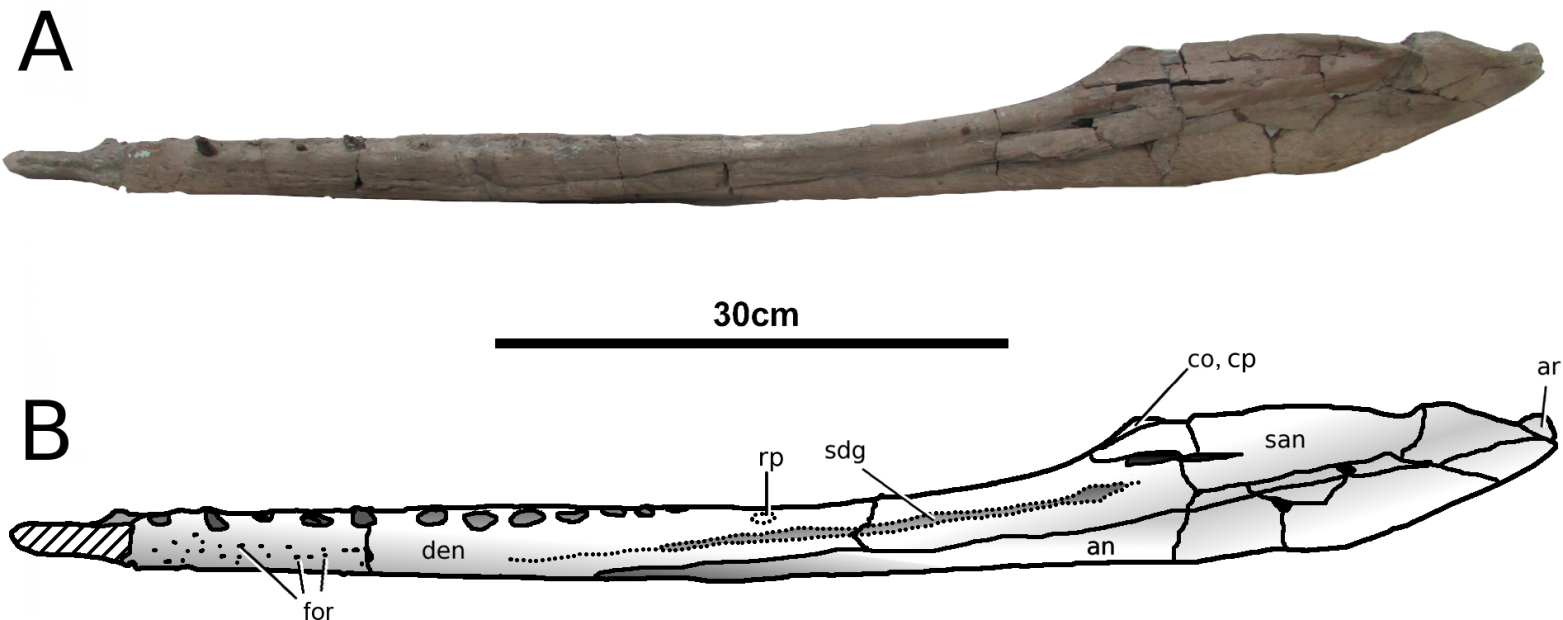
*Tyrannoneustes lythrodectikos*, PETMG:R60. Mandible. (A) Left lateral view photograph; (B) left lateral view line drawing. Refer to the main text for the abbreviations list.

**Figure 16:**
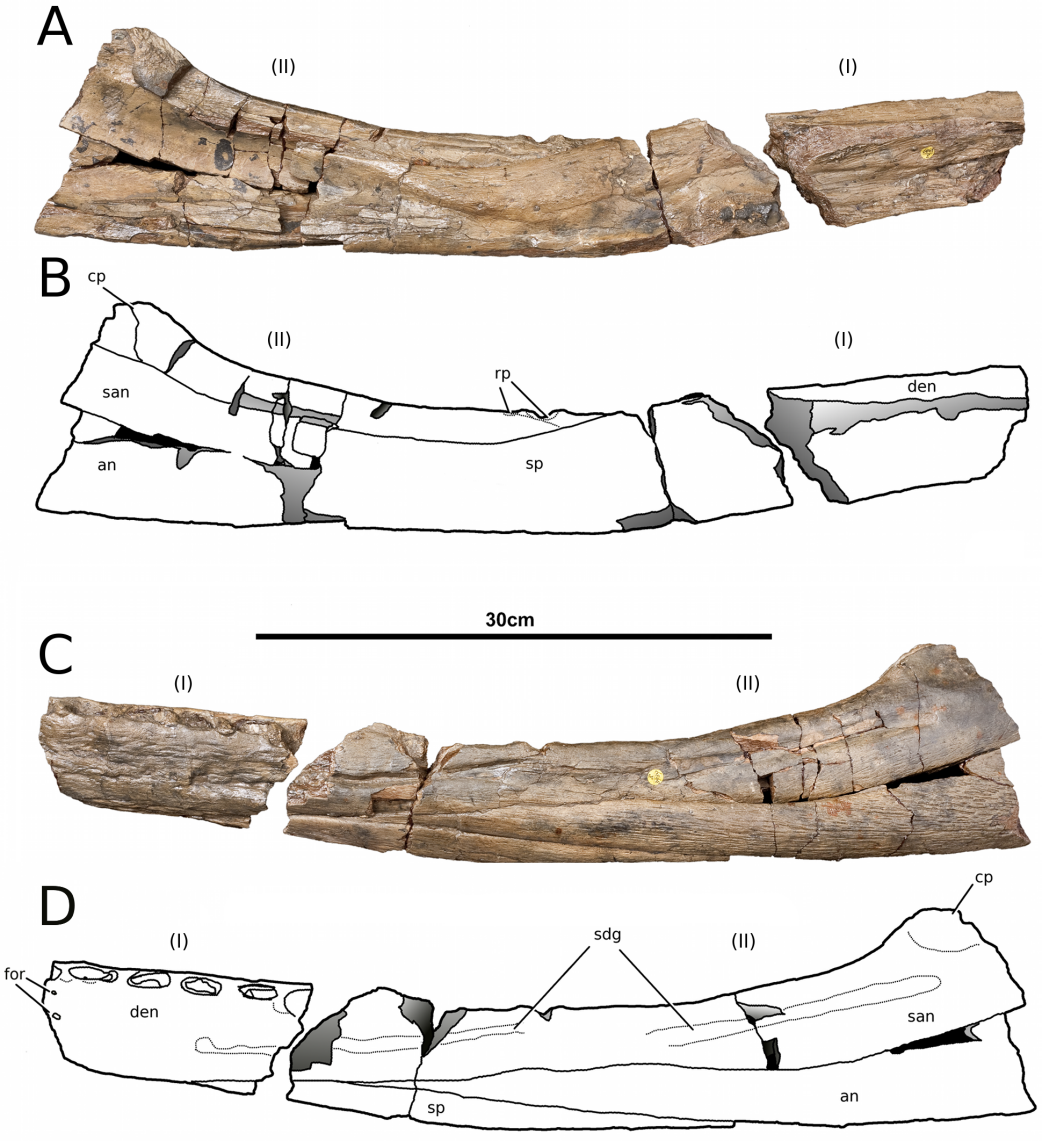
*Tyrannoneustes lythrodectikos,* NHMUK PV R3939. Mandible, left ramus. (A) Medial view photograph; (B) medial view line drawing; (C) lateral view photograph; (D) lateral view line drawing. Refer to the main text for the abbreviations list.

**Figure 17:**
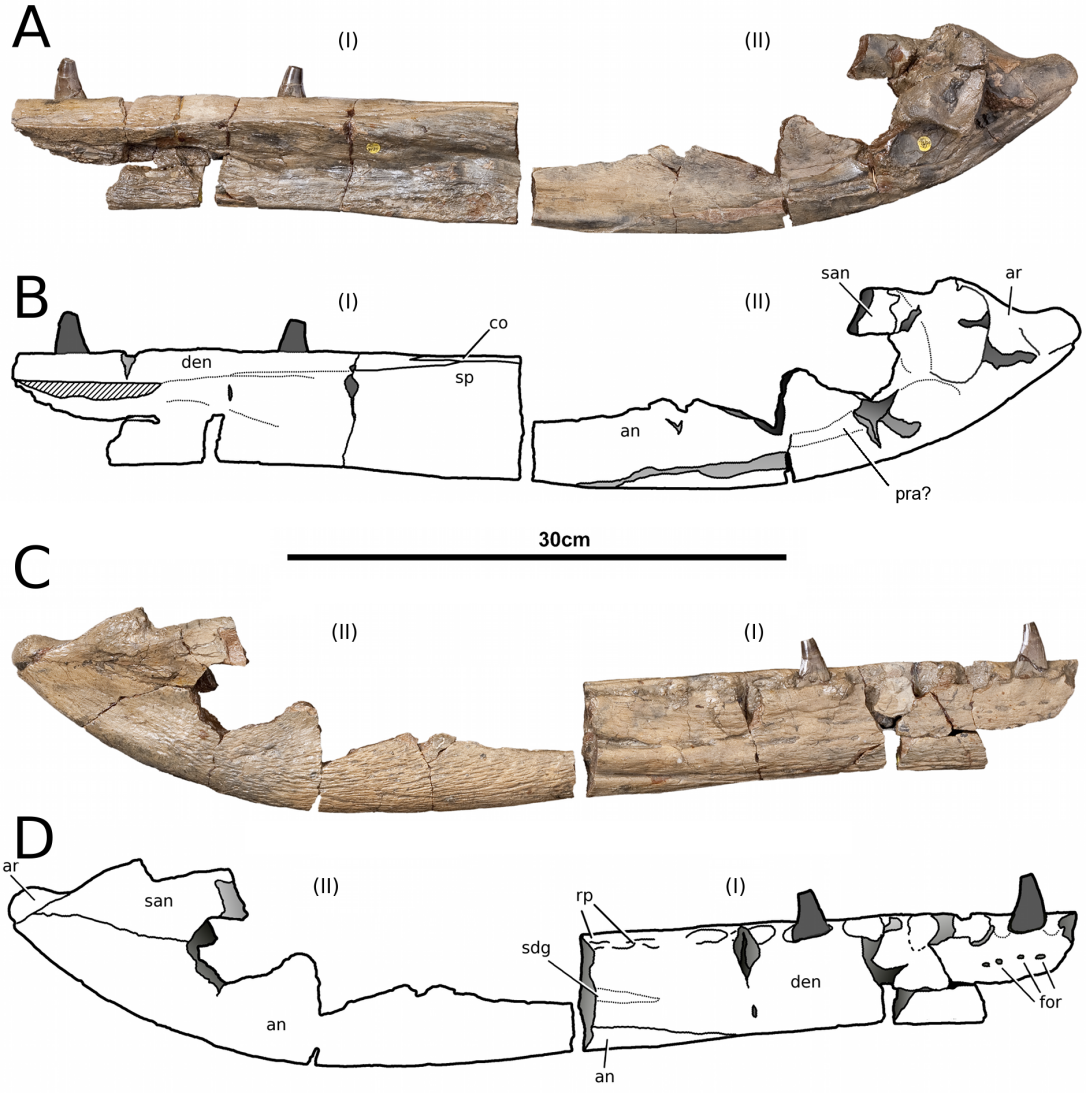
*Tyrannoneustes lythrodectikos*, NHMUK PV R3939. Mandible, right ramus. (A) Medial view photograph; (B) medial view line drawing; (C) lateral view photograph; (D) lateral view line drawing. Refer to the main text for the abbreviations list.

**Figure 18:**
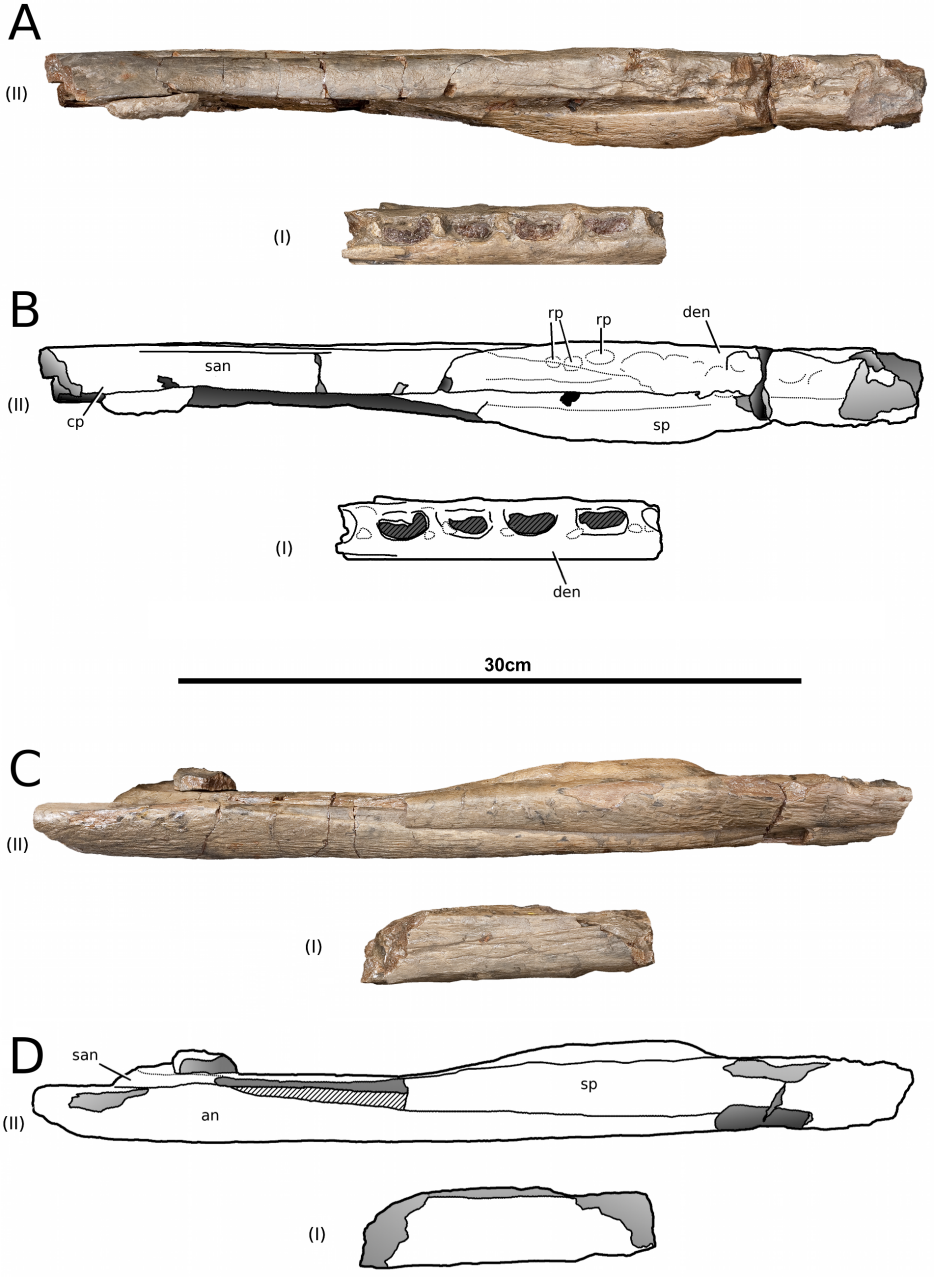
*Tyrannoneustes lythrodectikos*, NHMUK PV R3939. Mandible, left ramus. (A) Dorsal view photograph; (B) dorsal view line drawing; (C) ventral view photograph; (D) ventral view line drawing. Refer to the main text for the abbreviations list.

From the Marnes de Dives Formation (Bénerville-sur-Mer, département du Calvados, Basse-Normandie, France; upper Callovian): incomplete skull. Part of the Joaquim Gameiro private collection, see Lepage et al. (2008).

**Cf. *Tyrannoneustes* teeth.** Young et al. (2013a) referred isolated tooth crowns from the Callovian-Oxfordian of England, France and Poland to cf. *Tyrannoneustes*.

**Emended diagnosis.** Metriorhynchid crocodylomorph with the following unique combination of characters (autapomorphic characters are indicated by an asterisk): very long posterior process of the premaxilla, terminating level to the 4th or 5th maxillary alveoli^∗^; the first maxillary tooth pair is partially supported by the premaxilla and maxilla^∗^; distinct ‘notches’ on the lateral surface of the maxillae (in the anterior and middle regions)^∗^; 18 or 19 maxillary teeth, of which 10–11 are anterior to the palatine. The inflexion point of the prefrontal lateral margin (in dorsal view) is directed posteriorly at an angle less of 70 degrees or less from the anteroposterior axis of the skull; acute angle between the medial and the posterolateral processes of the frontal. The lateral margins of the postorbitals are significantly more lateral than the lateral-margins of the prefontals. 12–16 dentary teeth, 10–12 of which are adjacent to the mandibular symphysis. Reception pits for the maxillary teeth present posterior to the seventh dentary alveolus and continue posteriorly even after the dentary tooth row terminates; these reception pits are in the same plane as the dentary alveoli (or are on the posterior margin of the dentary alveoli) creating a mesiodistal in-line interlocking occlusion mechanism for the posterior-half of the tooth rows^∗^. Dentary tooth-row and coronoid process are ventrally displaced relative to the jaw joint (increased gape). The tooth crowns are moderately enlarged and strongly mediolaterally compressed. Very poorly defined microscopic true denticles present on both carinal keels, although the denticles do not proceed along the entire carina. In labial or lingual view the height of the denticles does not influence the height of the keel (i.e., no evident/conspicuous serrated edge). Enamel on the labial and lingual surfaces lack conspicuous ornamentation except at the base of the crown where there are accessory ridges orientated to the apicobasal axis of the crown^∗^; these ridges are low, widely-spaced and very short^∗^. Humerus shaft contributes more than 50% of total humeral length, proximal articular margin not in close contact with the deltopectoral crest, posterior margin of the humerus in lateral view distinctly concave. The deltopectoral crest is well-developed with the width of the humerus distal articular head is subequal to the width that the deltopectoral crest projects out from the humerus shaft. The ilium in lateral view: the dorsal margin of the articulation facet that contributes to the acetabulum is horizontally oriented^∗^. The dorsal border of the ilium is short, in lateral view it terminates posterior to the articulation facet that contributes to the acetabulum^∗^. Well-defined crest on the medial margin of the ischium between the articulation facet on the anterior process and the acetabulum^∗^ (modified from Young et al., 2013a).

**Body length estimate.** The skull of PETMG:R176 has a basicranial length of ∼86 cm, and the associated lower jaw PETMG:R60 has a length of 93 cm (matching the basicranial length of 85.3 cm reported by both Adams-Tresman, 1987; Vignaud, 1995). Using the Young et al. (2011) equations, this basicranial length results in an estimated total body length of 4.65 m. The French private specimen (Lepage et al., 2008) has a basicranial length of 84 cm, which would give an estimated total body length of 4.54 m.

However, NHMUK PV R3939 is an even larger individual than PETMG:R176 and PETMG:R60. As the skull is broken and incomplete, we used the ratio of basicranial length to interorbital distance in PETMG:R176 to estimate a basicranial length of ∼93 cm for NHMUK PV R3939 (this assumes that the ratio is interspecifically constant). This results in an estimated total body length of 5.04 m. Interestingly, the lack of vertebral neurocentral fusion suggests that NHMUK PV R3939 was not a morphologically mature individual (see discussion). CAMSM J64267 is from a smaller individual, comparable in size with the holotype GLAHM V972.

## Description and Comparisons

**SKULL**

**Premaxilla and external nares.** In NHMUK PV R3939 the premaxillae have partially collapsed along the midline premaxilla-premaxilla suture (Fig. 1). However, despite being damaged in the same area, in PETMG:R176 their natural shape can be discerned. In dorsal view, the premaxillae are mediolaterally enlarged with strongly curved lateral margins around the external nares (Fig. 2). In lateral view the external surface is slightly convex (Figs. 3 and 4). In both the specimens the external surface of the premaxillae are ornamented with a grooved and ridged pattern which appears to be orientated anteroposteriorly.

PETMG:R176 shows that *Tyrannoneustes lythrodectikos* has three round, widely spaced, premaxillary alveoli (Figs. 5 and 7A). In NHMUK PV R3939 only the posterior-most pair of alveoli are preserved (Fig. 6). When seen in dorsal view, the premaxillary posterior processes are very elongate, terminating approximately level to the 4th (M4) or 5th (M5) maxillary alveoli. The left and right premaxilla-maxilla sutures are gentle concave curves which converge posteriorly. The shape of this suture is variable among metriorhynchids, where it can be almost straight (‘V’-shaped), like in *Torvoneustes carpenteri* and *Cricosaurus lithographicus* (Wilkinson, Young & Benton, 2008; Herrera, Gasparini & Fernández, 2013), or concave as in *Dakosaurus andiniensis* (Pol & Gasparini, 2009) and *Maledictosuchus riclaensis* (Parrilla-Bel et al., 2013). In PETMG:R176 there is no premaxilla-nasal contact, as in most metriorhynchids except for *Cricosaurus macrospondylus* (Hua et al., 2000) and one specimen of ‘*Metriorhynchus*’ *brachyrhynchus* (NHMUK PV 3700, Andrews, 1913).

The external nares are better preserved in PETMG:R176 than in NHMUK PV R3939, which only preserves the posterior region. The external nares fossae are preserved on both premaxillae, and are deep concave ‘basins’. They are sub-triangular, anteroposteriorly elongated, and separated along the premaxillary midline by a raised region; however, due to preservation, we cannot ascertain whether they are separated into two distinct ‘basins’. This morphology is seen in all metriorhynchids (e.g., Eudes-Deslongchamps, 1867–1869; Fraas, 1902; Andrews, 1913; Pol & Gasparini, 2009; Young et al., 2010). In rhacheosaurin metriorhynchids this raised region is the posterior bar of the internarial bar (e.g., Fraas, 1901; Fraas, 1902; Gasparini & Dellapé, 1976; Hua et al., 2000; Herrera, Gasparini & Fernández, 2013; Parrilla-Bel et al., 2013). The posterior margin of these fossae terminates approximately in the same plane as the 3rd premaxillary alveoli (P3).

In palatal view (Figs. 5, 6 and 7A) the premaxilla-maxilla contact is a long and thin ‘V’-shape created by the maxilla palatal anterior process overlapping the premaxilla. The maxillary palatal process reaches level to the 2nd premaxillary alveoli (P2), and possibly extends even further anteriorly. The incisive foramen is preserved in front of the maxillary anterior processes (Figs. 5 and 7A), a feature rarely seen or mentioned for thalattosuchians. The contact participates to the 1st maxillary alveolar (M1) pair (Figs. 5, 6 and 7A).

**Maxilla.** In NHMUK PV R3939 and PETMG:R176 the maxillae are well preserved (Figs. 1–6). The external surfaces of the maxillae are slightly convex and strongly ornamented. There are three ornamentation patterns: (1) deep anteroposteriorly aligned grooves and raised ridges on the dorsal surface, (2) the ridges become more anastomosed in pattern posteriorly, adjacent to the maxilla-nasal suture, and (3) anteriorly and laterally, there is also an anastomosed pattern, but on the lateral surface closer to the tooth row the maxillae become smooth. Maxillary nutrient foramina occur along the lateral surfaces, but are better preserved on PETMG:R176 and the right maxilla of NHMUK PV R3939 (Figs. 3C, 4A and 4C). In both specimens they are elliptical with the long axis orientated anteroposteriorly. Unlike *Dakosaurus*, *Tyrannoneustes lythrodectikos* lacks enlarged foramina ventral to the preorbital fenestrae/fossae (Pol & Gasparini, 2009; Young et al., 2012b).

In NHMUK PV R3939 the maxillae are broken at the 12th (right) and 13th (left) maxillary alveoli, with the posterior skull region (the orbitotempotal region) preserving the contacts with the jugals (Fig. 8). The maxillary contact with lacrimal cannot be easily discerned in either NHMUK PV R3939 or PETMG:R176. As with the premaxilla, the maxilla is anteroposteriorly elongated, and it is as wide as it is deep, conferring tubular proportions to the rostrum. The sutural contacts with both the nasals and the premaxilla are concave curves. The lateral margins of the maxillae are concave when seen in dorsal view. This differs from the lateral margins of *Dakosaurus andiniensis* (Pol & Gasparini, 2009), *D. maximus* and *Plesiosuchus manselii* (Young et al., 2012b), which are almost straight or slightly convex. The preorbital fenestrae/fossae are not preserved because the posterior region of the maxillae are broken in NHMUK PV R3939 and have collapsed in PETMG:R176.

In lateral view, the ventral margins of the maxillae are weakly curved (Figs. 3 and 4). This results in the posterior maxillary teeth being ventral to the anterior maxillary and premaxillary teeth. This curvature of the tooth row is more pronounced in *Dakosaurus andiniensis* (Pol & Gasparini, 2009). Along the lateral margins, the ventral margins undulate due to distinct ‘notches’ (Figs. 3, 4 and 7). These notches are on the lateral surface of the maxillae, situated between the alveoli and only occur in the anterior and middle region of the maxillae. Interestingly, along the dorsal margin of these notches there are reception pits, most likely created by the dentary teeth (Figs. 3, 4, and 7D). These ‘notches’ are not seen in other metriorhynchids.

In NHMUK PV R3939 the ventral surfaces of the maxillae are sulcate, having two parallel anteroposterior sulci, one on either side of the midline (Fig. 6). These sulci appear to terminate at the level of 4th maxillary alveoli (M4). This feature has been previously described in *Maledictosuchus ricalensis* (see Fig. 8 in Parrilla-Bel et al., 2013). In the right maxilla of PETMG:R176 this feature is obliterated by post-mortem damage which pushed the palatal surface in a dorsal direction (Fig. 7). However, a sulcus is partially visible on the left maxilla from between the 11th and 7th maxillary alveoli. Anteriorly, and posteriorly, the sulcus is not present due to fractures/damages.

PETMG:R176 has 18–19 maxillary alveoli (the anterior 10–11 of which are anterior to the palatine-maxilla suture). Only 12 and 13 maxillary alveoli are preserved in the rostral part of NHMUK PV R3939; however in the orbitotemporal fragment an additional partial four alveoli are seen in the left maxilla (Fig. 8). Although the skull is fragmentary, and there is a gap between the rostral and orbitotemporal fragments, this specimen most likely also had 18–19 alveoli per maxilla. The inter-alveolar spacing is variable, with the posterior margin of the first maxillary alveoli (M1) and the anterior margin of second maxillary alveoli (M2) being the same thin lamina, whereas there are large inter-alveolar spaces between the alveoli from the M2 to the M6. Posterior to the M6, the inter-alveolar spaces become smaller. Irregular maxillary interalveolar spacing is also described in teleosaurids (Andrews, 1913), basal geosaurines (e. g. *‘Metriorhynchus’ brachyrhynchus*) and metriorhynchine metriorhynchids (Eudes-Deslongchamps, 1867–1869; Andrews, 1913). This morphology differs from the uniformly small maxillary inter-alveolar spaces seen in Geosaurini (Young et al., 2012b; Young, 2014). It is hypothesised that the uniformly small inter-alveolar spaces are related to the enlarged tooth crowns in this sub-clade (Young, 2014).

**Nasals.** Nasals are paired, unfused and large triangular/subtriangular elements. They suffered diagenetic fractures in NHMUK PV R3939 (Figs. 1, 3 and 9) but are relatively intact in PETMG:R176 (Figs. 2 and 7). The lateroposterior processes (a metriorhynchid apomorphy; Young et al., 2013b; Young, 2014) are not preserved in NHMUK PV R3939, while they are dorsoventrally compressed in PETMG:R176. The nasal dorsoposterior processes are intact in PETMG:R176, and are partially preserved in the orbitotemporal fragment of NHMUK PV R3939 (Figs. 2 and 9). The dorsoposterior processes contact the frontal medially (the anteromedial frontal process) and the prefrontal laterally. The nasals contact the anteromedial process of the frontal in an irregular way, as in *Gracilineustes leedsi*, *Metriorhynchus durobrivensis*, *Cricosaurus saltillensis* and *Maledictosuchus riclaensis* (Andrews, 1913; Buchy, Young & Andrade, 2013; Parrilla-Bel et al., 2013) rather than in a simple V-shaped contact as in *Metriorhynchus superciliosus* and *‘Metriorhynchus’ brachyrhynchus* (Andrews, 1913) (Figs. 2 and 9). Amongst metriorhynchids the shape of the nasal-prefrontal suture is variable, generally being almost straight or weakly concave; however, in the genus *Cricosaurus* this suture is curved with a posterolaterally orientated pronounced concavity (Andrews, 1913; Gasparini & Dellapé, 1976; Pol & Gasparini, 2009; Young et al., 2012b; Young et al., 2013a; Herrera, Gasparini & Fernández, 2013). In *T. lythrodectikos* this suture is straight and similar in morphology to the one of ‘*Metriorhynchus*’ *brachyrhynchus* (Andrews, 1913). The contact with the lacrimal is not preserved.

As with the other dermatocranial bones, the external surface of the nasals has a pronounced ornamentation. Numerous anteroposteriorly aligned grooves and raised ridges sculpt the nasals, but the pattern shifts along the surface. At the anterior processes, the ridged-grooved pattern is deep and pronounced. Posterior to the anterior processes, the ornamentation shifts to shorter ridges and grooves that are more widely distributed. Moreover, they are not all anteroposteriorly orientated. Adjacent to the nasal-maxilla sutural contact the ridges and grooves have a more anastomosed pattern. On the dorsoposterior process the ridged-grooved ornamentation is more tightly packed, is orientated anteroposteriorly, and is not deep or pronounced.

**Prefrontals.** The dorsal surfaces of the prefrontals are large and sub-triangular, that are almost complete in PETMG:R176 (Fig. 2), and poorly preserved in NHMUK PV R3939 (Fig. 9). The descending processes have suffered dorsoventral compression in both specimens making an accurate description impossible.

The dorsal surfaces of the prefrontals are laterally enlarged and overhang the anterior third of the orbit (a metriorhynchid synapomorphy; Eudes-Deslongchamps, 1867–1869; Fraas, 1902; Andrews, 1913). The external surface is slightly convex and is covered in large ridges and grooves arranged in an anastomosed pattern. In dorsal view, the prefrontals contact the nasals anteromedially and the frontal posteromedially. The nasal-prefrontal suture is almost straight, while the frontal–prefrontal suture is a strongly convex curve. The prefrontal posterior margins form the anterior margin of the supraorbital notches. The posterodorsal edge of the prefrontals forms a strong convexity, with an inflexion point of approximately 70° compared to the anteroposterior axis of the skull (similar to *Plesiosuchus manselii, Torvoneustes carpenteri, Torvoneustes coryphaeus,* and *Geosaurus grandis*; Wilkinson, Young & Benton, 2008; Young & Andrade, 2009; Young et al., 2012b; Young et al., 2013b). This strongly differs from *Dakosaurus*, where this angle is approximately 50° (Pol & Gasparini, 2009; Young et al., 2012b). In ‘*Metriorhynchus*’ *brachyrhynchus* however, instead of the lateral and posterior margins of the prefrontal forming a convex curve, they abruptly meet in an approximately 90° angle (NHMUK PV R3699, NHMUK PV R3700, NHMUK PV R3804; Andrews, 1913). In comparison with other Oxford Clay Formation taxa, *Tyrannoneustes lythrodectikos* prefrontals are proportionally larger, particularly in having greater lateral extension (Fig. 10).

**Lacrimals.** These bones are only partially preserved and are severely damaged. In both NHMUK PV R3939 and PETMG:R176 the dorsoventral compression of the preorbital areas, and the dorsal displacement of the jugal bars and posterior processes of the nasals, make it impossible to describe the contacts of the lacrimal with the surrounding elements.

**Jugals.** The morphology of the jugals, their sutural contacts and participation/exclusion from the preorbital fossae and fenestrae cannot be certainly assessed. Only a small fragment of the left jugal of NHMUK PV R3939 is anteriorly preserved, while the right jugal is broken, rotated and displaced from its original position (Figs. 3 and 9). In PETMG:R176 both the jugal bars have been dorsally displaced but while the right is largely missing, the left one is substantially intact although its posterior section is hidden behind the broken ventral process of the postorbital (Figs. 2, 4 and 5). The jugal bar has a slight dorsal concavity and sub-triangular cross section as opposed to the elliptic-circular cross-section of *Geosaurus giganteus* (Young & Andrade, 2009), and the rectangular cross-section of *Maledictosuchus riclaensis* (Parrilla-Bel et al., 2013). The jugal anterior process contacts with the posterior process of the maxilla, with the maxilla being ventral to the jugal.

**Postorbitals.** Only the left postorbital of PETMG:R176 is complete (Figs. 2 and 4). The right postorbital of PETMG:R176 and both postorbitals of NHMUK PV R3939 are missing in large parts, although each preserves the anterior process and its contact with the lateral process of the frontals (Figs. 2 and 9). The anterior process of the postorbital contacts the lateral process of the frontal in a ‘V’-shaped suture that is posterolaterally orientated (i.e., the frontal overlaps the postorbitals). At this suture there is an abrupt change in external surface ornamentation pattern. On the postorbital the external ornamentation is composed by a network of deep grooves. The postorbital contact with the squamosal is well preserved on both side of the skull in PETMG:R176 (Fig. 4). The descending process of the postorbital is largely missing, such that the entire anterior margin of the supratemporal fenestra is not preserved. The postorbital forms the majority of the supratemporal arch, forming the lateral rim of the supratemporal fenestra and the dorsal margin of the subtemporal fenestra. The postorbital posteriorly meets the squamosal with a straight and posterodorsally inclined suture.

The lateral extent and curvature of the anterior process of the postorbitals is considerable as in *Neptunidraco ammoniticus* (Cau & Fanti, 2011; Cau, 2014). The lateral margins of the postorbitals are in a sagittal plane that, proportionally, is noticeably lateral to the sagittal plane that passes through the lateral margin of the prefrontal (Fig. 10). As a consequence, the temporal region of *Tyrannoneustes lythrodectikos* would have been comparatively larger than in the contemporaneous ‘*Metriorhynchus’ brachyrhynchus, Metriorhynchus superciliosus* and*Gracilineustes leedsi* (Andrews, 1913). Increasing the volume of the supratemporal fenestrae would have important mechanical and soft-tissue consequences (i.e., large temporal adductor musculature muscle volume), and it would represent further evidence that many of the macrophagous adaptations evolved before Geosaurini (Wilkinson, Young & Benton, 2008; Pol & Gasparini, 2009; Young & Andrade, 2009; Andrade et al., 2010; Young et al., 2012b; Young et al., 2013a; Young et al., 2013b; Cau, 2014).

**Frontal.** The frontal is a single, fused, element. The external surface has no sign of an interfrontal suture, and is strongly ornamented by elliptical pits, grooves and ridges (Figs. 2, 4 and 9). On the central region of the frontal the grooves and ridges are tightly packed and become radially orientated towards the supraorbital notches and lateral processes of the frontal. These radial grooves and ridges are finer and shallower. On the anteromedial process there are numerous very small pits with occasional long ridges that are orientated to the anteroposterior axis of the skull. On the posterior process the elliptical pits become elongated grooves that are also orientated parallel to the anteroposterior axis of the skull.

The anteromedial process is broken in NHMUK PV3939 (Fig. 9), but it is intact in PETMG:R176 (Fig. 2). It does not reach as far anteriorly as the anterior margins of the prefrontals, as in *Geosaurus giganteus* and *Torvoneustes carpenteri* (Wilkinson, Young & Benton, 2008; Young & Andrade, 2009), but it is not as short as in *Cricosaurus lithographicus* (Herrera, Gasparini & Fernández, 2013). In *Torvoneustes coryphaeus*, *Cricosaurus araucanensis*, and *Dakosaurus andiniensis* the anterior margin of the anteromedial process either reaches level to the anterior margin of the prefrontals, or extends further anteriorly (Gasparini & Dellapé, 1976; Pol & Gasparini, 2009; Young et al., 2013a). In both NHMUK PV R3939 and PETMG:R176 the anteromedial process continues posterolaterally, contacting the prefrontals until the process reaches the supraorbital notches.

Both lateral processes of the frontal are posterolaterally curved. The lateral processes contact the postorbitals posteriorly. The angle between the frontal midline and the lateral processes is approximately 66°. This angle is highly variable in Metriorhynchidae from a minimum of ∼45° in *Dakosaurus* and *Geosaurus* (Pol & Gasparini, 2009), through intermediate values ∼50 in the rhachaeosaurines *Maledictosuchus riclaensis* (Parrilla-Bel et al., 2013) and *Cricosaurus saltillensis* (Buchy, Young & Andrade, 2013), ∼60° in the two *Torvoneustes* species (Wilkinson, Young & Benton, 2008; Young et al., 2013a) to the maximum of nearly 90 degrees in *Metriorhynchus superciliosus* (Andrews, 1913; Wilkinson, Young & Benton, 2008). The frontal posteromedial process, which forms the anterior margin of the intertemporal bar, is incomplete in NHMUK PV R3939 (Fig. 9) but is intact in PETMG:R176 (Fig. 2). Between the frontal lateral processes and the posteromedial process, the frontal enters the supratemporal fossae; however it is excluded from the supratemporal fenestrae by the parietal-squamosal contact (as in *Torvoneustes coryphaeus*; Young et al., 2013b).

**Proötic.** The proötics and the lateral region of the laterosphenoids can be seen within the supratemporal fenestrae in dorsal view for PETMG:R176. Unfortunately, damage and unprepared matrix make the sutures between the proötics and the laterosphenoids difficult to determine. In *Pelagosaurus typus*, *Metriorhynchus superciliosus*, ‘*M*.’ *casamiquelai, Cricosaurus araucanensis* and *Dakosaurus andiniensis* the proötics and laterosphenoids contact, and form a blunt dorsoventrally orientated crest (Fernández et al., 2011; MTY, pers. obs., 2014), which is present also in *Tyrannoneustes lythrodectikos*. The proötic normally contacts the squamosal (posteriorly) and the parietal (anteriorly). *Tyrannoneustes* proötic is triangular in shape in anterior-lateral view, it is slightly concave and appears to be comparatively much larger than in ‘*Metriorhynchus*’ *casamiquelai* (Fernández et al., 2011). Typically the proötics and laterosphenoids form the dorsal and anterior margin of the trigeminal fenestrae (Fernández et al., 2011; Young et al., 2012b); unfortunately the preservation of PETMG:R176 does not allow us to assess if this is the case for *Tyrannoneustes*.

**Laterosphenoid.** In PETMG:R176 the left laterosphenoid is better preserved than the right. The ventral margins of both laterosphenoids are severely damaged. The laterosphenoids are large, and form the majority of the medial and ventral part of the supratemporal fenestrae (Fernández et al., 2011; Young et al., 2012b). The laterosphenoids contact the parietal dorsally, the proötics posteriorly and would form the anterior margin of the trigeminal fenestrae. Again, cracks and unprepared matrix hide critical features and the sutural contacts of the psoterior section of the laterosphenoids.

**Parietal.** Only PETMG:R176 preserves the parietal which, in dorsal view, is a single T-shaped element which has no trace of an interparietal suture on its external surface (Fig. 2). Its lateral and anterior processes have suffered heavy diagenetic distortion, resulting in many missing fragments. As with other thalattosuchians, the parietal contributes to the medial margin of the supratemporal fenestrae along with the posterior process of the frontal and the proötics (e.g., Andrews, 1913). The parietal-frontal contact is ‘M-shaped’ in dorsal view (Fig. 2) and morphologically not significantly different from ‘*Metriorhynchus*’ *brachyrhynchus* and *Maledictosuchus riclaensis* (Parrilla-Bel et al., 2013). However in *Tyrannoneustes lythrodectikos*, the parietal anterior process develops more further anteriorly than in other two taxa. i.e., it terminates more anteriorly (Fig. 2). The proötics (diagenetically damaged) and quadrates can be seen within the supratemporal fossae of PETMG:R176, where they form the medioposterior and posterolateral margins (Fig. 2). The parietal contact with the squamosals cannot be properly described due to damage of the posterior temporal area.

**Squamosals.** The squamosals are only preserved in PETMG:R176 (Figs. 2 and 4). Unfortunately, both experienced diagenetic damage, and their dorsal margins are partially eroded. They form the posterolateral margin of the supratemporal fossa and their lateral process articulates with the postorbitals. A long ventral process projects anteriorly giving the squamosal-postorbital contact a concave profile in lateral view. In posterior view, the squamosal contacts the quadrate ventrally, the exoccipital-opisthotic complex ventrolaterally and the parietal dorsally (Fig. 11). The precise morphology of these sutures cannot be assessed due to the poor conditions of the occipital surface.

The posterolateral surface of the squamosal bears a large, smooth, slightly concave surface, which is a common feature among metriorhynchids; having been reported in *Cricosaurus araucanensis, Cricosaurus lithographicus*, *Maledictosuchus riclaensis*, *Torvoneustes coryphaeus* and *Dakosaurus andiniensis* (Pol & Gasparini, 2009; Herrera, Gasparini & Fernández, 2013; Parrilla-Bel et al., 2013; Young et al., 2013b). The squamosal extends vertically as a smooth straight surface forming the greatest part of the dorsal wall of the supratemporal fossa and fenestra. It articulates with the quadrate with a horizontal suture.

**Quadrates.** The quadrates in PETMG:R176 have undergone strong deformation resulting in them being rotated and dorsoventrally compressed. The quadrate contacts the squamosals and the opisthotics dorsally and the exoccipital medially. However, these sutures are not clearly visible due to extensive damage and matrix on the occipital surface (Fig. 11). The quadrate articular surface has two pronounced convex condyles divided by a shallow fossa. These surfaces articulate with the matching opposite morphology of the articular glenoid fossae.

**Supraoccipital.** The supraoccipital is a single element on the skull midline which is exposed on the occipital surface (Fig. 11). In PETMG:R176 all that is preserved of the foramen magnum is the ventral margin, and the slightly damaged dorsal margin. The supraoccipital has been described in a few metriorhynchids as a single rhomboidal-subhexagonal or dorsoventrally elongated element, adjoining the parietal (dorsally, sometimes dorsolaterally) and the exoccipital (laterally and occasionally ventrally). In *Cricosaurus araucanensis*, ‘*Metriorhynchus*’ *westermanni* and ‘*Metriorhynchus*’ cf. *M. westermanni*, and *Plesiosuchus manselii* the ventral margin of the supraoccipital forms the dorsal margin of the foramen magnum (Gasparini & Dellapé, 1976; Gasparini, Paulina-Carabajal & Chong, 2008; Fernández et al., 2011; Young et al., 2012b). *Tyrannoneustes lythrodectikos*, like *Maledictosuchus riclaensis, Cricosaurus schroederi, ‘Metriorhynchus’ brachyrhynchus, ‘Metriorhynchus’* cf. *durobrivensis*, *Metriorhynchus superciliosus,* and *Dakosaurus andiniensis* have the supraoccipital excluded from the foramen magnum by the exoccipitals (Karl et al., 2006; Lepage et al., 2008; Pol & Gasparini, 2009; Young et al., 2012b; Parrilla-Bel et al., 2013). Unfortunately, the diagenetic damage to the occipital surface means the supraoccipital–exoccipital/opisthotic complex suture cannot be seen.

**Exoccipital-opisthotic complex.** The exoccipital-opisthotic complex appears to be a single unit (the otoccipital), however there could be a suture ventral to the paroccipital processes (Fig. 11) which suggests they may be separate elements (a similar suture is seen in *Torvoneustes coryphaeus*; Young et al., 2013b). This complex is the main ‘element’ seen on the occipital surface, and forms the lateral and dorsal margins of the foramen magnum. The dorsolateral part of their surface is concave. The paroccipital processes are only slightly damaged but most likely rotated from their original position. Between the exoccipital and the quadrates (immediately ventral to the paroccipital processes and the potential exoccipital-opisthotic suture) there is a continuous fracture. It probably represents the line around which the quadrates articular processes rotated during diagenesis.

A number of foramina are visible along the lateral margin of the exoccipital, being lateral to the foramen magnum and basioccipital condyle (Fig. 11). In life these most likely hosted cranial nerves and blood vessels. Based on comparisons with *Dakosaurus andiniensis, Maledictosuchus riclaensis, Cricosaurus araucanensis,* ‘*Metriorhynchus*’ *westermanni* ‘*Metriorhynchus*’ cf. *M. westermanni* and *Torvoneustes coryphaeus* (Gasparini & Dellapé, 1976; Gasparini, Paulina-Carabajal & Chong, 2008; Fernández et al., 2011; Parrilla-Bel et al., 2013; Young et al., 2013b; Herrera & Vennari, in press) the largest and most ventral pair of foramina corresponds to those for the internal carotids. Dorsal to these there are three smaller foramina corresponding to those for the X, XI and XII cranial nerves (Fig. 11).

**Basioccipital.** The basioccipital is the main element forming the occipital condyle, which in *Tyrannoneustes lythrodectikos* is wider than high (Fig. 11). There is a pit on the posterior surface of the occipital condyle as in *Dakosaurus andiniensis* (Pol & Gasparini, 2009). At the dorsolateral corners of the occipital condyle there are two sutures, each of which is the contact between the exoccipitals and the basioccipital, as in *Plesiosuchus manselii* (Young et al., 2012b).

Ventral to the occipital condyle there are two ventrally-orientated processes, the basioccipital tuberosities (i.e., the basal tubera). The tubera are separated by a deep intertuberal notch within which is the medial eustachian foramen. The ventral surfaces of the tubera are partially worn/eroded (Figs. 5, 7B and 7C).

**Basisphenoid.** The basisphenoid is a triangular shaped element of which only the ventral surface is preserved. It is posterolaterally confined by the basioccipital tuberosities. Its ventral surface has thin crests forming a ‘Y-shape’ and delimiting three fossae, of which the lateral two are elongated and teardrop-shaped while the posterior-most (and the smallest) is triangular in shape (Fig. 7C). Similar converging crests are also seen in *Cricosaurus lithographicus* and *Maledictosuchus ricalensis* (see Figs. IC and 1D in Herrera, Gasparini & Fernández, 2013; Parrilla-Bel et al., 2013), and may be a common feature among thalattosuchians. Unfortunately, direct comparison is impossible for many taxa, as the basisphenoid is typically poorly preserved, much like the pterygoids and ectopterygoids (e.g., ‘*Metriorhynchus*’ *westermanni*, ‘*Metriorhynchus*’ cf. *M. westermanni*, *Dakosaurus* spp., *Geosaurus* spp., *Plesiosuchus manselii* (Gasparini, Paulina-Carabajal & Chong, 2008; Pol & Gasparini, 2009; Fernández et al., 2011; Young & Andrade, 2009; Young et al., 2012b)).

**Palatines.** The palatines are almost entirely missing in NHMUK PV R3939, with only the anterior-region of the midline anterior process preserved (Fig. 6). However, they can be confidently described in PETMG:R176 despite the palatal surface being partly collapsed and the posterior section being poorly preserved (Figs. 5 and 7D). The palatine maxillary contact is a narrow semi-lunate curve extending far as the 10–11th maxillary alveoli.

Palatine morphology is quite diverse in Metriorhynchidae, with different subclades having different morphologies. In Metriorhynchinae, the palatines have two non-midline anterior processes (Andrews, 1913; Gasparini & Dellapé, 1976; Lepage et al., 2008; Young et al., 2012b), while the rhacheosaurin *Maledictosuchus riclaensis* apomorphically has two non-midline anterior processes and one midline process (Parrilla-Bel et al., 2013). Geosaurines, including *Tyrannoneustes lythrodectikos*, have a single midline anterior process (Andrews, 1913; Young et al., 2012b). This is most likely the plesiomorphic condition, as basal metriorhynchoids like *Teleidosaurus calvadosii* and teleosaurids share the single midline anterior process (Eudes-Deslongchamps, 1867–1869; Andrews, 1913; Lepage et al., 2008).

**Pterygoids.** No specimens preserve the pterygoids. Moreover, the internal nares and the posterior region of the palatines are also not preserved.

**MANDIBLE**

**Preservation and general morphology.** CAMSM J64267 is preserved in good condition (Fig. 12), and is 626 mm in length. This means that it is from a smaller individual than the holotype, which although incomplete, measured approximately 670 mm (Young et al., 2013a). In PETMG:R60 the mandibular rami are almost completely intact, only missing the anterior part of the symphyseal region (Figs. 13–15). However, the posterior part of each ramus has been severely distorted, and slightly damaged on the medial side. Unfortunately, the two rami have been wrongly aligned and glued together along the symphyseal suture (Figs. 13–15). Consequently the right ramus is misaligned in the anteroposterior direction by one tooth position (∼4–5 cm). Evidence for this misalignment is based on: (1) the anterior ends of the splenials and coronoids not being level (Figs. 13 and 14), and (2) the relative position of the diastema between D4 and D5 in the left and right rami.

**Figure 19:**
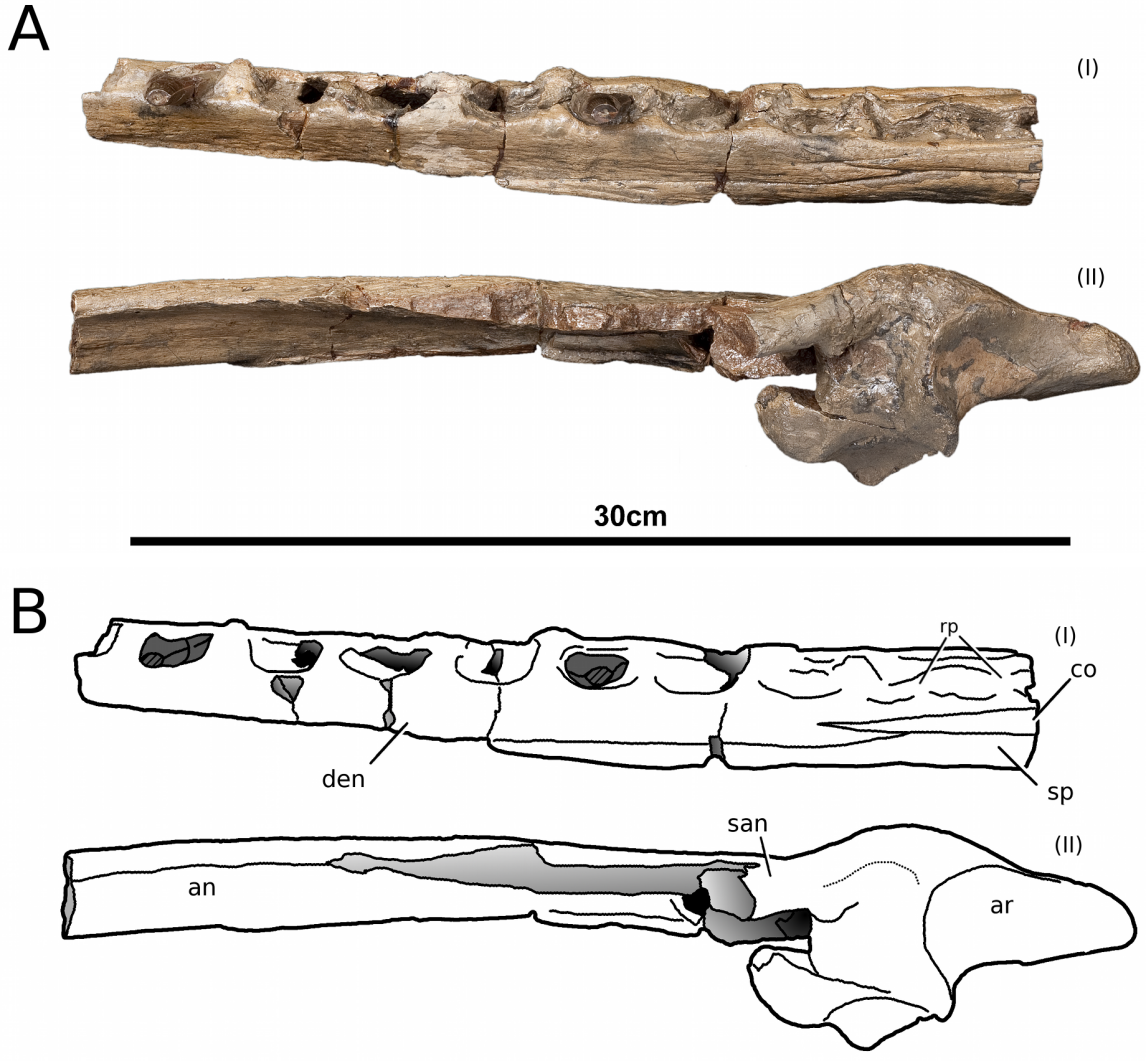
*Tyrannoneustes lythrodectikos*, NHMUK PV R3939. Mandible, right ramus. (A) Dorsal view photograph; (B) dorsal view line drawing. Refer to the main text for the abbreviations list.

**Figure 20:**
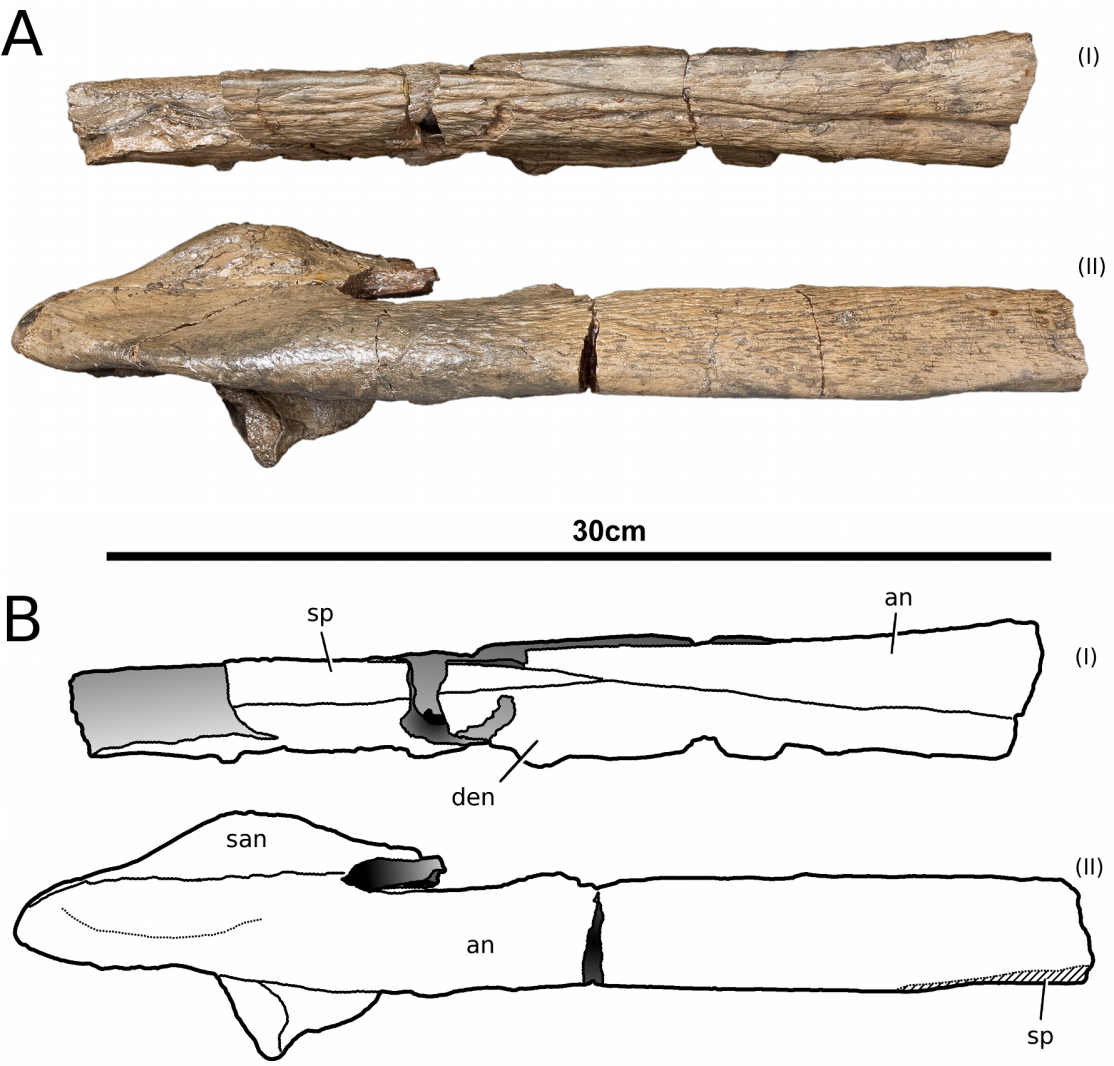
*Tyrannoneustes lythrodectikos*, NHMUK PV R3939. Mandible, right ramus. (A) Ventral view photograph; (B) ventral view line drawing. Refer to the main text for the abbreviations list.

In NHMUK PV R3939 both rami are incomplete and broken in two pieces which do not fit together (Figs. 16–20). However, comparison with the complete rami of the holotype CAMSM J64267 and PETMG:R60 allows us to estimate the positions of both the anterior pieces. In particular the tooth count, the absence of preserved D4–D5 diastema, the position of the surangulodentary suture, the presence/absence of foramina and the position of the coronoid suggests that: (1) the anterior fragment of NHMUK PV R3939 right mandibular ramus (I in Figs. 17, 19 and 20) broke anteriorly at the 5th dentary alveolus and posteriorly behind the posterior-most alveoli; the second fragment (II in Figs. 17, 19 and 20), which is posterior to the tooth row, preserves the angular, the articular, a small portion of the surangular, and a possible fragment of the prearticular; (2) The anterior fragment of the left ramus (I in Figs. 16 and 18) must belong to the middle section for the lower jaws. The posterior fragment (II in Figs. 16 and 18) goes from the back tooth row to the coronoid process.

**Figure 21:**
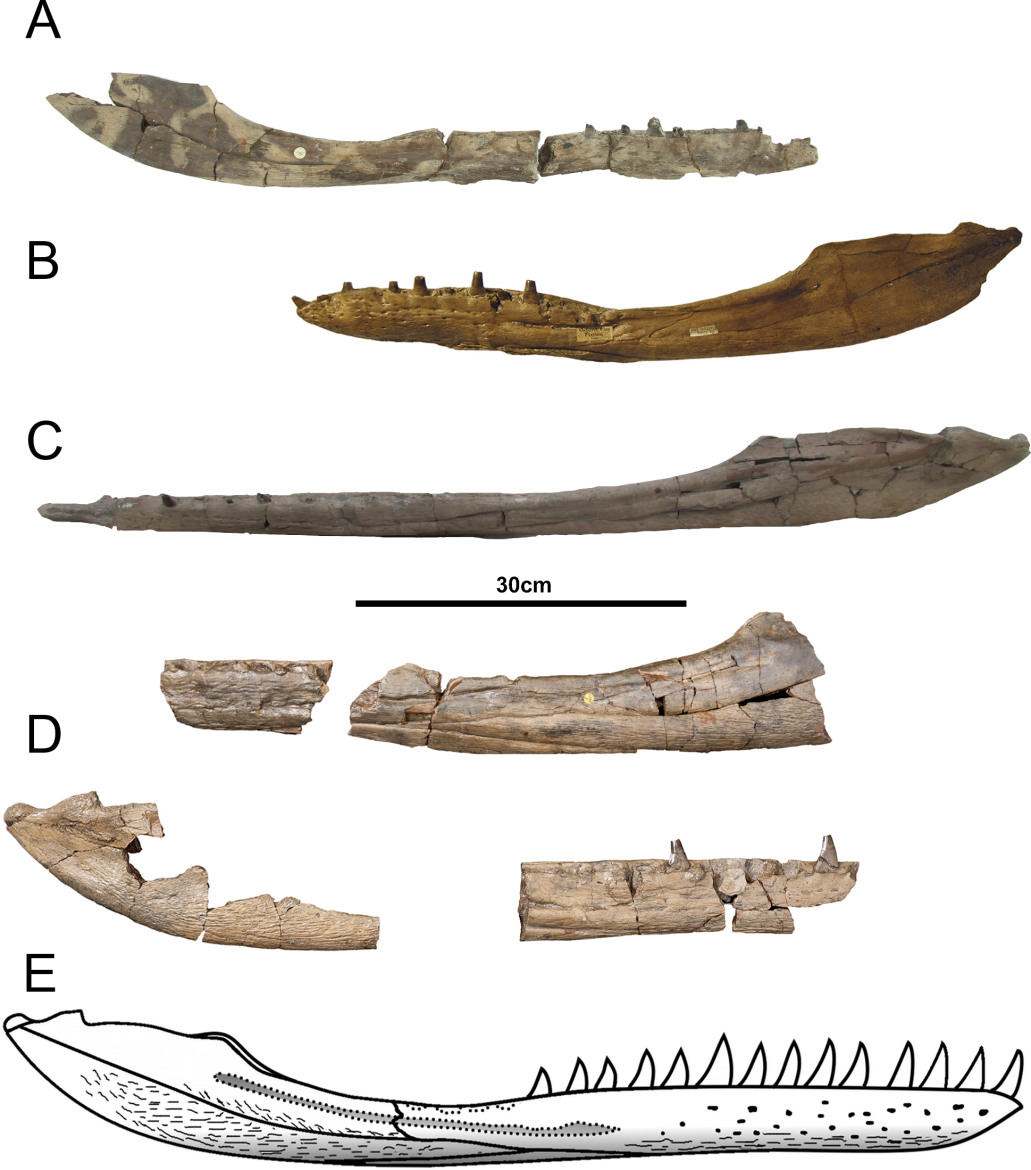
*Tyrannoneustes lythrodectikos* comparative mandibular rami, lateral views. (A) GLAM V972, right mandibular ramus; (B) CAMSM J64267 left mandibular ramus; (C) PETMG:R60, left mandibular ramus; (D) NHMUK PV R3939, left and right mandibular rami; (E) left ramus schematic reconstruction.

In the subclade Geosaurini, the genera have a deep and well-defined marked surangulodentary groove on the lateral surface of the rami (Pol & Gasparini, 2009; Young & Andrade, 2009; Young et al., 2012a; Young et al., 2013b). In NHMUK PV R3939, PETMG:R176, CAMSM J64267 and the holotype of *Tyrannoneustes lythrodectikos* (Young et al., 2013a) this groove is shallower and less defined (Figs. 12–15, 16C, 16D, 17C, 17D, and 21). In *Dakosaurus*, there is a large foramen at the dentary terminus of this groove (Pol & Gasparini, 2009; Young et al., 2012b). No large foramen is present in NHMUK PV R3939, while we cannot ascertain its presence in PETMG:R60 due to post-mortem deformation of the mandibular rami. There is a small shallow foramen at the surangular terminus of the surangulodentary groove in the *Tyrannoneustes lythrodectikos* holotype (Young et al., 2013a), CAMSM J64267 (Fig. 12A) and NHMUK PV R3939 (Figs. 16C and 16D).

**Dentaries.** The anterior region of the dentary has a small number of medium-sized foramina which are located approximately mid-way between the tooth row and the ventral margin (Figs. 12, 14, 15, 17C, 17D and 21). This is distinct from the numerous large foramina seen in the anterior dentaries of *Dakosaurus* (Young et al., 2012b). The ornamentation pattern on the external surface of the dentaries in CAMSM J64267, NHMUK PV R3939 and PETMG:R60 is not homogeneous across the element: along the ventral margin there is, in fact, a pronounced grooved-ridged pattern, whereas closer to the tooth row the surface is largely smooth.

Among thalattosuchians a diastema occurs between the 4th and 5th dentary alveoli (Eudes-Deslongchamps, 1867–1869; Andrews, 1913; Hua et al., 1993; Lepage et al., 2008; Young et al., 2013b; Nesbitt et al., 2013). This feature is absent in the Geosaurini genera *Dakosaurus* and *Plesiosuchus* (Young et al., 2012b) and in *Neptunidraco* sp. (Cau, 2014). This diastema is present in the holotype of *T. lythrodectikos* (GLAHM V972). The jaw fragments of NHMUK PV R3939 lack the anterior-most part of the mandibular symphysis, thus the diastema is not preserved. Comparing the holotype with PETMG:R60 and CAMSM J64267 suggests that the diastema is preserved also in larger, presumably ontogenetically more mature specimens.

In the holotype of *T. lythrodectikos,* PETMG:R60 and CAMSM J64267 the mandibular symphysis is long, between one third and half of total mandibular length. This is similar to the condition seen in Geosaurini, but considerably shorter than long snouted metriorhynchids such as *Gracilineustes leedsi* (Andrews, 1913; Young et al., 2012b). The mandibular symphysis of *T. lythrodectikos* is also moderately deep. It has been suggested that metriorhynchids with large tooth crowns, for example the geosaurins *Dakosaurus* and *Plesiosuchus* also have deep symphyses (Young et al., 2012b).

In NHMUK PV R3939, PETMG:R60 and CAMSM J64267 the sutures between the dentary and the angular and the splenial are clearly visible, and match the gently dorsally curving morphology common in metriorhynchids (Figs. 12, 14, 15, 16C, 16D and 21). Unlike the holotype (GLAHM V972) and PETMG:R60 (Fig. 5E), the dentary-surangular contact cannot be clearly seen in NHMUK PV R3939; the partial fusion of this suture could be due to preservation or ontogeny (Figs. 12, 14, 15, 16A, 16B and 21).

**Splenials.** The entire posterior part of the right splenial, and part of the left, are missing in NHMUK PV R3939. The medial posterior surfaces of the mandibular rami are distorted and severely damaged in PETMG:R60, consequently the splenial-surangular contact cannot be confidently described. This region of CAMSM J64267 is crushed, resulting in large cracks which partially obscures the suture (Fig. 12A). However, the middle and anterior parts of the splenials are well enough preserved to show that they match the splenial description of the holotype (Young et al., 2013a). In all the specimens the dorsal surface broadly follows the dorsal margin of the mandible outline in lateral view but it forms a strong concavity just behind the tooth row. The splenials contribute largely to the ventral surface of each mandibular ramus, and are involved in the posterior part of the symphyseal suture. Young et al. (2013a) could not quantify the extent of this contribution due to the extent of post-mortem damage that the anterior region of the holotype GLAHM V972 experienced. In both dorsal and ventral view of PETMG:R60, the splenial anterior process reaches level to the 8th dentary alveoli. This contribution is as significant as in other basal geosaurines (e.g., ‘*Metriorhynchus’ brachyrhynchus*) (Andrews, 1913) and it is more extensive than in the meso-longirostrine Metriorhynchinae (e.g., *Gracilineustes* spp., *Rhacheosaurus* spp., *Maledictosuchus ricalensis*) (Andrews, 1913; Young et al., 2010; Parrilla-Bel et al., 2013). Conversely, the shortening of the snout in Geosaurini may have resulted in the splenial being comparatively more involved in the midline mandibular suture on both ventral and dorsal surfaces (Pol & Gasparini, 2009; Young et al., 2012b).

**Angulars and surangulars.** Both elements are only partially preserved in each ramus of NHMUK PV R3939, and they are distorted in both PETMG:R60 and CAMSM J64267. The surangular-angular contact is a gently curved suture running for nearly half of the mandibular ramus. Its anterior end is a weakly concave line starting from a triple contact with the dentary which becomes almost straight just before the coronoid process. The suture terminates at the articular contact. The angular occupies the ventral part of the posterior half of the lower jaw and its ventral margin follows a strong upward bend and rising to the posterior end of the mandible above the coronoid process. Similarly, the concave curvature of the angular dorsal surface in lateral view becomes gradually more pronounced approaching the coronoid process as in the holotype of *T. lythrodectikos* (GLAHM V972) (Figs. 12, 15 and 16). This geometric shift in mandible shape readily distinguishing *T. lythrodectikos* from other metriorhynchids. The angulars and surangulars of *T. lythrodectikos* are somewhat intermediate in robusticity between the stouter and deeper elements of Geosaurini (Wilkinson, Young & Benton, 2008; Pol & Gasparini, 2009; Young et al., 2012b) and the more gracile condition seen amongst most metriorhynchines (Andrews, 1913; Herrera, Gasparini & Fernández, 2013; Parrilla-Bel et al., 2013).

**Coronoids.** The right coronoid is intact in PETMG:R60 and CAMSM J64267, while the left coronoids in both NHMUK PV R3939 and PETMG:R60 are missing, but their shape and anterior extension can be confidently determined by a sulcus for the element on the dorsomedial surface of the of the left rami. As in other thalattosuchians, the coronoid fits between the surangular and splenial (posteriorly) and the dentary and splenial (anteriorly). Overall the coronoid appears as a thin, rod like element following the dorsal surangular curvature. It makes a weak ‘S’-shape due to it curving dorsolaterally immediately posterior to the tooth row; anterior to this, it projects anteriorly as a thin straight element with a tapering end that intrudes into the dentary. The coronoid does not participate in the external surface of the mandible or in the mandibular symphysis. Just like the holotype, the coronoid extends as far as the end of tooth row (12th alevoli). In *Tyrannoneustes lythrodectikos* and Geosaurini the coronoid is always ventral to both glenoid fossa and retroarticular process (Young et al., 2012b; Young et al., 2013a).

**Articulars.** Both of the articulars are preserved in PETMG:R60 and CAMSM J64267, with the right articular preserved in NHMUK PV R3939. They also preserve the retroarticular processes and the glenoid fossae, all of which were missing in the holotype (Fig. 21). As in *Torvoneustes coryphaeus* and ‘*Metriorhynchus*’ *brachyrhynchus* (Andrews, 1913; Young et al., 2013b), the articular surface for the quadrate consists of two concave surfaces; the posterior being created by the curvature of the retroarticular process, while the anterior-most is the glenoid fossa, hosting the quadrate in life. A low anterodistally oriented ridge subdivides the fossa into concavities, as in *Torvoneustes coryphaeus* but not *Plesiosuchus manselii* (Young et al., 2012b; Young et al., 2013b). However, unlikely *Torvoneustes*, the glenoid fossa is approximately as long as it is wide (although it is damaged). The retroarticular process is posterior to the glenoid fossa.

The raised ridge separating the retroarticular process from the glenoid fossa resembles that seen in *Torvoneustes coryphaeus*, in that it is not straight but is a sinusoidal curve, concave medially and convex laterally (Young et al., 2013b). In dorsal view, the retroarticular process has a sub-triangular shape, as in other metriorhynchids (Andrews, 1913; Gasparini & Dellapé, 1976; Herrera, Gasparini & Fernández, 2013; Young et al., 2013b). The retroarticular process is longer than wide (narrower than the glenoid fossa), with a weakly curved lateral margin and a posteriorly straight medial one.

**Dentition**

**Figure 22:**
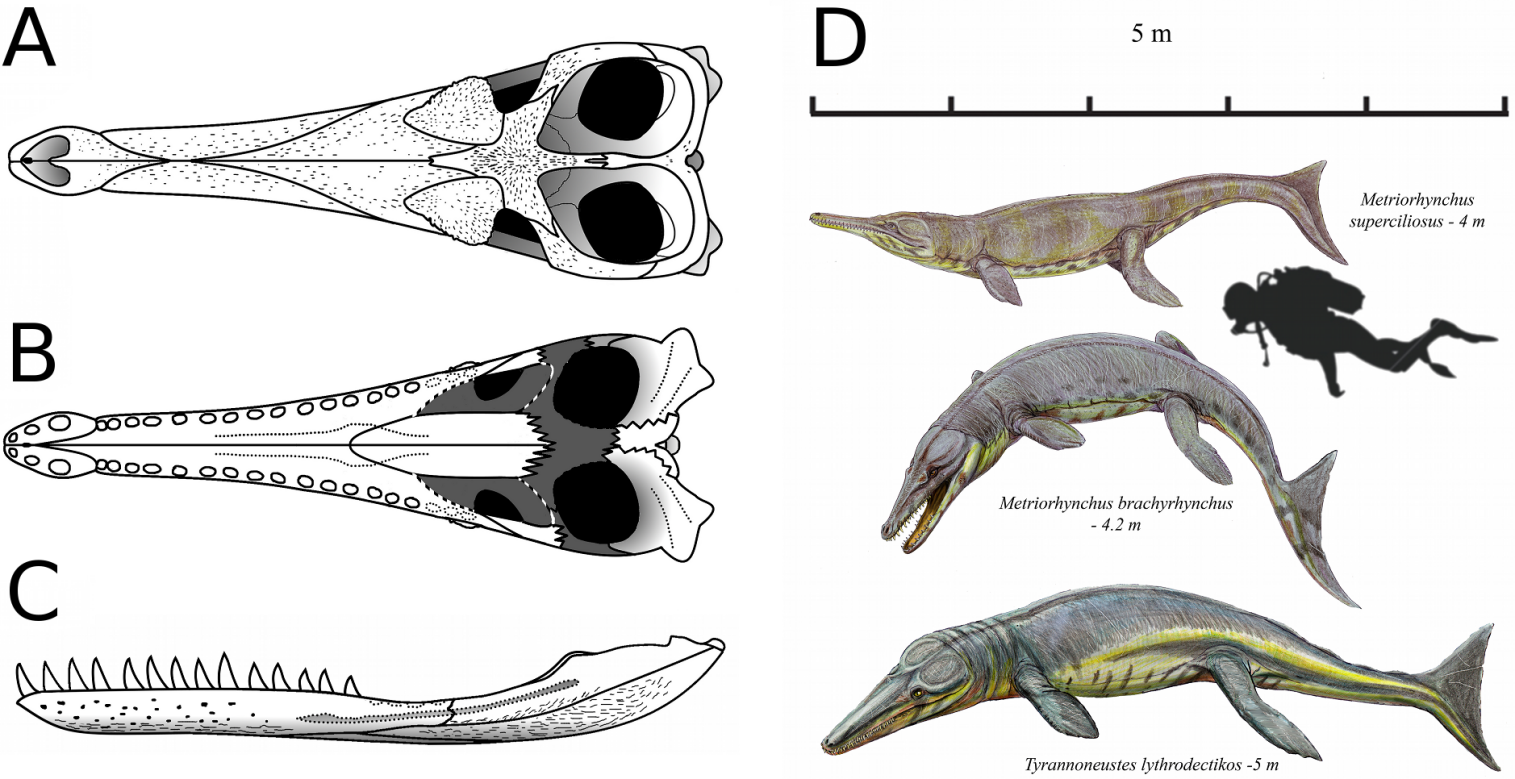
*Tyrannoneustes lythrodectikos*. Skull schematic reconstruction and life reconstruction. (A) Dorsal view; (B) palatal view; (C) lower jaw schematic reconstruction, lateral view; (D) Oxford Clay Formation metriorhynchids (courtesy of and by Dmitry Bogdanov).

**Tooth count and morphology.** Based on PETMG:R176, the *Tyrannoneustes lythrodectikos* skull had three premaxillary teeth, and approximately 18–19 maxillary teeth (Figs. 5, 7A and 22). The poor preservation of the posterior-most maxillary alveoli makes the maxillary tooth count uncertain. The skull of NHMUK PV R3939 is incomplete, with the rostrum broken in line with the last premaxillary alveoli (Fig. 6). The anterior skull fragment has 12 alveoli per side preserved, with 3 or 4 poorly preserved alveoli along the right maxillary process (seen in the orbitotemporal fragment) (Figs. 6 and 8). Considering the gap between the two skull pieces, it is highly likely the total maxillary count was similar to that of PETMG:R176.

Both dentaries of PETMG:R60 preserve at least 14 alveoli (Fig. 13). The posterior-most sections are poorly preserved, which makes it difficult to distinguish between alveoli, damage and receptions pits (just like the holotype; Young et al., 2013a). However, posterior to the 14th dentary alveoli there would only be enough space for one or two more alveoli (Fig. 13). In the smaller specimen CAMSM J64267 there are 12 definitively preserved alveoli, with space for up to two more posteriorly (Fig. 12). As such, we estimate there to be a minimum of 12 alveoli per dentary, but possibly up to 16 in some specimens. Young et al. (2013a) could not be sure whether there was 12 or 14 dentary alveoli preserved in the holotype, due to the similarity of the posterior-most alveoli and the reception pits. Based on PETMG:R176, CAMSM J64267 and the holotype (GLAHM V972) there are 10–12 alveoli adjacent to the mandibular symphysis. As previously noticed, the preservation of the lower jaws in NHMUK PV R3939 prevents a precise tooth count, but the longest jaw fragments have 8–9 complete alveoli and two incomplete alveoli. In the holotype (GLAHM V972) the D1 alveolus is not as strongly procumbent as in longirostrine forms (e.g., Parrilla-Bel et al., 2013) or orientated so that the first tooth crown was directed immediately out of the mouth as in *Dakosaurus* (Young et al., 2012a). The dorsoventral compression of the anterior symphyseal region of PETMG:R60 makes it look like the D1–D2 alveoli were more procumbent than it actually would have been (Fig. 15). In both GLAHM V972 and CAMSM J64267 the D1 alveoli are slightly orientated anterodorsally (Figs. 21A and 21B).

When compared to other Oxford Clay metriorhynchid species, *Tyrannoneustes lythrodectikos* has a lower tooth count than the metriorhynchines *Metriorhynchus superciliosus* and *Gracilineustes leedsi*, but had a similar tooth count to the basal geosaurinae ‘*Metriorhynchus*’ *brachyrhynchus* (see Young et al., 2013a).

**Figure 23:**
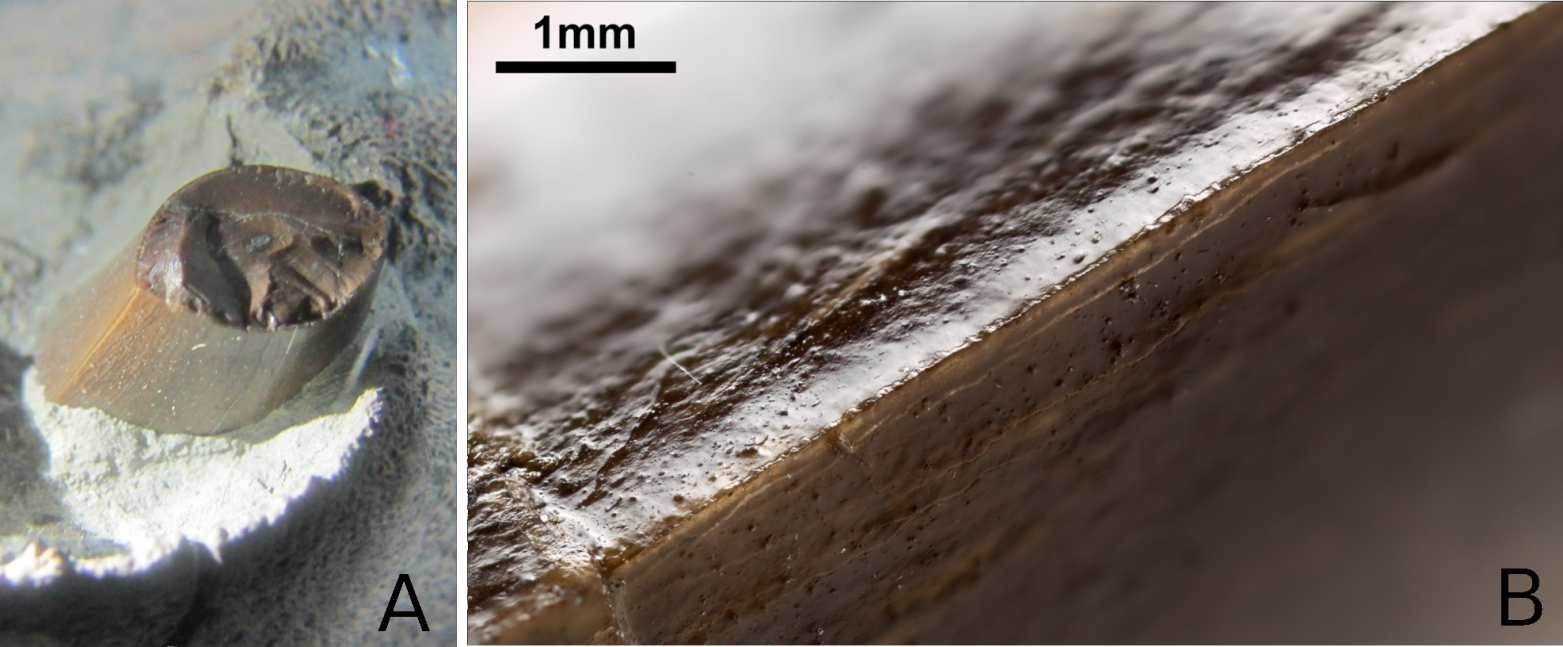
*Tyrannoneustes lythrodectikos*, dentition. (A) PETMG:R176 maxillary tooth; (B) NHMUK PV R3939, close up of a tooth.

**Ornamentation, carinae and wear.** The holotype, CAMSM J64267, NHMUK PV R3939, PETMG:R176 and PETMG:R60 preserve in situ tooth crowns. Unfortunately, very few are intact, as most are broken near the level of the dentigerous bone surface. As noted by Young et al. (2013a), the tooth crowns of *Tyrannoneustes lythrodectikos* are intermediate in apicobasal length between the subclade Geosaurini and basal geosaurines/metriorhynchines. The teeth are bicarinate, and like the holotype (GLAHM V972) the carinae are formed by a keel with microscopic, very poorly developed non-contiguous denticles (Fig. 23). The dentary and maxillary teeth are strongly mediolaterally compressed (somewhat like ‘*Metriorhynchus*’ *brachyrhynchus*; Young et al., 2013a). The enamel ornamentation is composed of low-relief apicobasal ridges, which are visible in the holotype (Young et al., 2013a) and PETMG:R176 (Fig. 23), but in NHMUK PV R3939 they are not clearly visible, most likely due to the generous amount of consolidant applied during the restoration process.

## Discussion

### *Tyrannoneustes lythrodectikos* overview

The holotype of *Tyrannoneustes lythrodectikos* was based on an incomplete skeleton of a sub-adult (Young et al., 2013a). While some bone fragments may have belonged to the skull, the mandible, ilium and ischium of this individual all had diagnostic characteristics. Here we described two/three much larger individuals (NHMUK PV R3939, PETMG:R176 and PETMG:R60), and one smaller individual (CAMSM J64267). While the mandibular rami of NHMUK PV R3939 are incomplete and damaged, and those of PETMG:R60 and CAMSM J64267 are distorted, all specimens are very similar to the holotype (GLAHM V972) (Table 1). In particular, the sharply rising surangular, and the gently rising angular and the ventral displacement of the coronoid process with respect of the articular surface. Posteriorly on the dentary, there are reception pits at the posterior end of the dentary tooth row. The preservation of the dentary alveoli and the interalveolar spaces preclude us from determining if they continued as far anteriorly as the 7th dentary alveolus (like the holotype; Young et al., 2013a). However, the reception pits are clearly visible on both the left and right rami. As with the holotype, these reception pits are in the same plane as the dentary alveoli, which creates an in-line interlocking dentition for the posterior-half of the tooth rows. This occlusion mechanism is unique to *Tyrannoneustes lythrodectikos* (Young et al., 2013a).

Some mandibular differences can be seen between the sub-adults (the holotype and CAMSM J64267) and the two larger specimens (NHMUK PV R3939 and PETMG:R60). For instance, they are considerably larger and more robust than GLAHM V972 and CAMSM J64267. The external ornamentation on the angulars and surangulars in all the specimens have the same pattern, and only varies in how ‘pronounced’ they are (they are more prominent in NHMUK PV R3939, PETMG:R176 and PETMG:R60). These differences can be attributed to intraspecific variation, sexual dimorphism and/or ontogeny.

The skulls of NHMUK PV R3939 and PETMG:R176 share three autapomorphic characteristics: (1) very long posterior process of the premaxilla (which terminates level to the 4th or 5th maxillary alveoli); (2) distinct ‘notches’ on the lateral surface of the maxillae (in the anterior and middle regions) which have reception pits on the dorsal margin and (3) the first maxillary alveoli are partially supported by the premaxillae and maxillae. The presence of these ‘notches’ in the anterior and middle region of the maxillary tooth row is very interesting. It shows that the occlusion mechanism is different along the tooth row (which also occurs in the Callovian metriorhynchine *Maledictosuchus ricalensis*; Parrilla-Bel et al., 2013). In the anterior/mid region there are vertically orientated tooth crowns which, when the jaws were closed, would have been in slightly different sagittal planes (i.e., the dentary tooth row would have been slightly lateral to the premaxillary–maxillary tooth row) (evidenced by the ‘notches’ and the reception pits on their dorsal margins). In contrast, in the posterior region the vertically orientated tooth crowns would have been in the same sagittal plane when the jaws closed (as evidenced by the in-line reception pits on the dentary). The presence of vertically orientated tooth crowns which are arranged so that during adduction they come close, or contact, is a feature of two Geosaurini genera: *Dakosaurus* and *Geosaurus* (Pol & Gasparini, 2009; Young & Andrade, 2009; Young et al., 2012a). As such, the evolution of shearing occlusion mechanics probably evolved prior to Geosaurini.

Among the Oxford Clay Formation taxa, the most (superficially) similar taxon to *Tyrannoneustes lythrodectikos* is ‘*Metriorhynchus*’ *brachyrhynchus.* This superficial similarity explains why CAMSM J64267, NHMUK PV R3939 and PETMG:R176 have been overlooked for so long. However, Andrews’ (1913) referral of NHMUK PV R3939 to ‘*Metriorhynchus*’ *brachyrhynchus* was only tentative. Overall cranial shape is similar between *T*. *lythrodectikos* and ‘*M*.’ *brachyrhynchus*, as is the pronouncement of the dermatocranial ornamentation, dental morphology and tooth counts. However, detailed examination of CAMSM J64267, NHMUK PV R3939 and PETMG:R176 shows that mandibular shape is radically different from ‘*Metriorhynchus*’ *brachyrhynchus* (Young et al., 2013a). As is the position of the reception pits on the dentary (Young et al., 2013a), presence of ‘notches’ on the lateral surface of the maxilla, the elongated premaxillary posterior processes, the shape of the prefrontals, different ratio of interorbital distance to basicranial length (0.13 for *T. lythrodectikos* and 0.16 for ‘*M*.’ *brachyrhynchus*) and the enlarged supratemporal fenestrae. This shows that the similarities in craniomandibular morphology between ‘*Metriorhynchus*’ *brachyrhynchus* and *Tyrannoneustes lythrodectikos* are superficial, and they do indeed represent distinct taxa.

**Table 1:** Table of diagnostic characters for Metriorhynchidae, and various subclades, for *Tyrannoneustes lythrodectikos* specimens from the Callovian. Table is updated from Young et al. (2013a).

Clades	Diagnostic characters	GLAHM V972	NHMUK PV R3939	PETMG: R60	CAMSM J64267
Metriorhynchidae	Absence of mandibular fenestrae	Yes	Yes	Yes	Yes
	Coronoid process on mandible	Yes	Yes	Yes	Yes
Metriorhynchid symplesiomorphies	Variable maxillary interalveolar spaces (from more than half of adjacent alveolar size to smaller)	?	Yes	Yes	Yes
	Variable dentary interalveolar (less than half the size of the immediate alveoli)	Yes	?	Yes	Yes
	Tubular snout (not broad, with width and depth being sub-equal)	?	Yes	Yes	?
	Maxillary alveoli count is 17 or more	?	Yes	Yes	?
Geosaurinae	Denticulated bicarinate dentition	Yes	Yes	?	?
Geosaurine symplesiomorphies	Not contiguous denticles along the carinae	Yes	Yes	?	?
*Tyrannoneustes* + Geosaurini	Ventral displacement of the dentary tooth row, such that the coronoid process is located considerably above the plane of the tooth row	Yes	?	Yes	Yes
	Dentary alveoli count is 16 or fewer	Yes	?	Yes	Yes
*Tyrannoneustes*	Reception pits on the dentary, normally in the sagittal plane between alveoli 7 and 12, or the reception pits are on the posterior margin of the dentary alveoli	Yes	Yes	Yes	Yes
	Mandible geometry: the curvature of the mandible ventral surface in lateral view. The convex curvature at the dentary-angular suture and anterior region of the angular is very gentle, and does not become pronounced until level to the coronoid process. After which the convex curvature becomes large and pronounced.	Yes	Yes	Yes	Yes
	Mandible geometry: the curvature mandible dorsal surface in lateral view. The dentary-surangular suture and anterior region of the surangular forms a large and marked concave curve.	Yes	Yes	Yes	Yes

### NHMUK PV R3939 is a large but not a morphologically mature individual of *Tyrannoneustes lythrodectikos*

**Figure 24:**
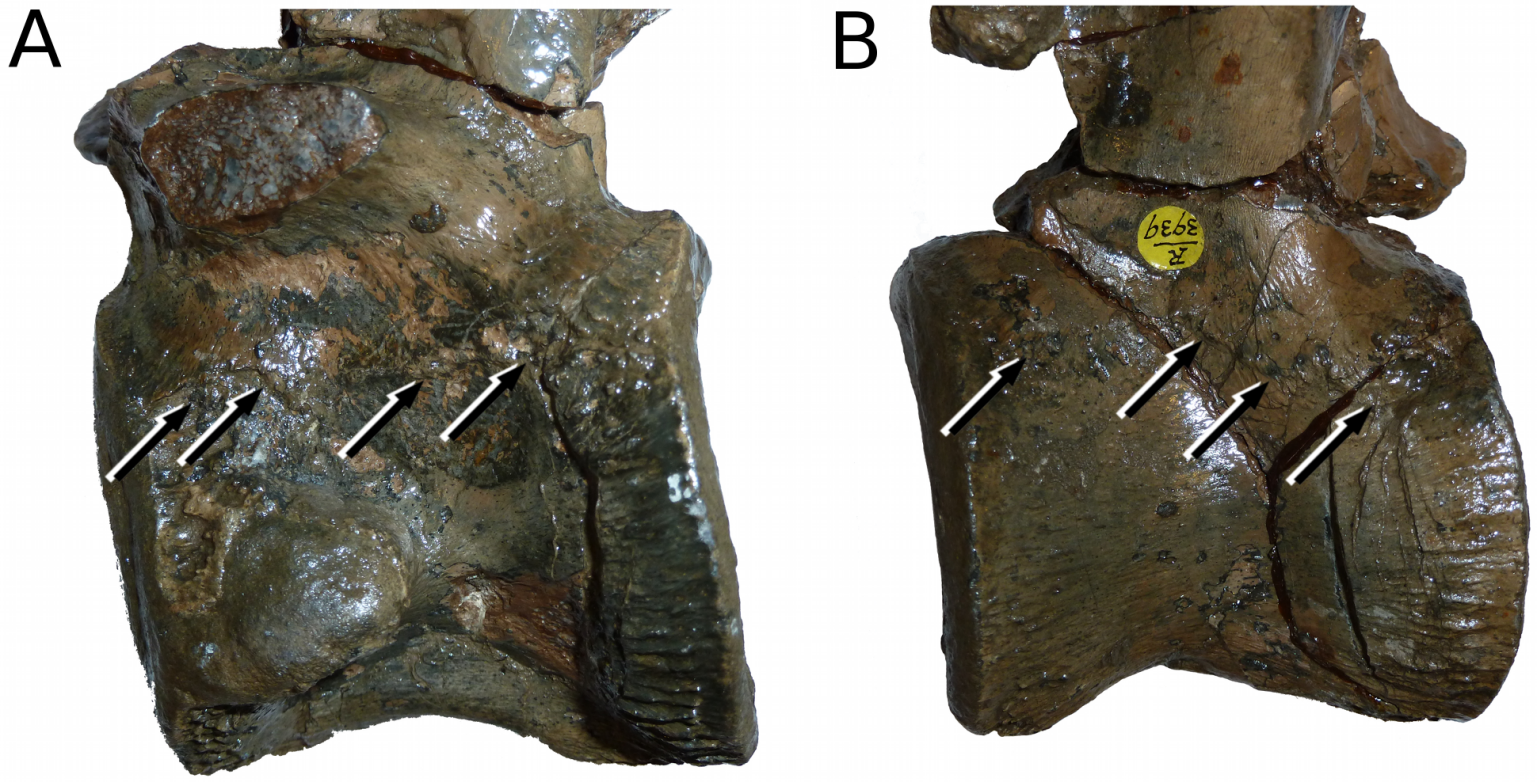
*Tyrannoneustes lythrodectikos*, NHMUK PV R3939. Vertebrae. (A) Dorsal vertebra, left lateral view; (B) cervical vertebra, right lateral view. Arrows indicate the incompletely fused neurocentral sutures.

The degree of closure for the neurocentral sutures along the vertebral column have often been used as an indicator of ontogentic stage in both extant and extinct crocodylomorphs (e.g., Brochu, 1996; Delfino & Dal Sasso, 2006; Herrera, Fernández & Gasparini, 2013b; Young et al., 2013a). The suture closure pattern follows a regular unidirectional progression from caudal to cervical vertebrae, the latter ones being completely fused only in morphologically mature specimens, sometimes after sexual maturity (Brochu, 1996; Ikejiri, 2012). The *T. lythrodectikos* holotype shows that the same sequence of neurocentral fusion occurs in metriorhynchids (Young et al., 2013a). Moreover, examination of ‘*Metriorhynchus’ brachyrhynchus* (NHMUK PV R3804) and *Metriorhynchus superciliosus* (NHMUK PV R 2054) vertebral columns show that neurocentral fusion occurs in all vertebrae in large specimens. Two of the *Tyrannoneustes lythrodectikos* specimens have vertebrae preserved, the holotype (GLAHM V972) has 33 vertebrae (4 cervical, 9 dorsal and 20 caudal vertebrae), and only the caudals show complete neurocentral fusion. NHMUK PV R3939 preserves only four dorsal and four cervical vertebrae, none of which show neurocentral suture fusion (Fig. 24). As a consequence neither individuals should be considered skeletally mature; however the larger size, the different degree of bone ornamentation and the partial fusion of surangulodentary suture, suggests that at least NHMUK PV R3939 was morphologically more mature than the holotype (GLAHM V972).

### Evolution of wide-gape macrophagy in Geosaurinae

Within Geosaurinae there is a trend towards wide-gape macrophagy (= the capability to feed on larger bodied prey items than their phylogenetic sister taxa; *sensu* Young, Steel & Middleton, 2014). This macroevolutionary trend begins with the Bajocian-Bathonian taxon *Neptunidraco ammoniticus* (and *Neptunidraco* sp.) (Cau & Fanti, 2011; Cau, 2014). There is currently no consensus as to whether the macrophagous adaptations seen in *Neptunidraco* and *Tyrannoneustes* + Geosaurini are convergent evolution or evidence of a deep, shared, origin of macrophagy in Geosaurinae (Young et al., 2013a; Young et al., 2013b; Cau, 2014). Only new discoveries from the Bajocian and Bathonian can answer this question.

All of the Oxford Clay Formation metriorhynchids (*Tyrannoneustes lythrodectikos*, *Metriorhynchus superciliosus*, ‘*Metriorhynchus*’ *brachyrhynchus* and *Gracilineustes leedsi*) have some degree of dentary tooth row ventral displacement (created by the geometric re-arrangement of the posterior mandible; see Young et al., 2013a) (Table 2). However, as noted above, the strong curvature of the posterior mandible in *Tyrannoneustes lythrodectikos* (the holotype GLAHM V972, CAMSM J64267, NHMUK PV R3939 and PETMG:R60) readily distinguishes it from these other taxa. This ventral displacement of the dentary tooth row results in a greater ‘optimal’ gape (*sensu* Young et al., 2013a). The increase in optimal gape and tooth crown apicobasal length in *Tyrannoneustes lythrodectikos* is suggestive of feeding on larger-bodied prey. One of the problems with feeding at wider gapes is that biting performance is reduced (Herring & Herring, 1974; Dumont & Herrel, 2003; Bourke et al., 2008). This may be the evolutionary impetus for predatory taxa evolving adaptations for higher biting performances at wider gapes (e.g., Herring & Herring, 1974; Young et al., 2013a). This leads us to ask, which macrophagous adaptations *Tyrannoneustes* shared with Geosaurini, and which it did not.

**Table 2:** Table of data derived from the optimum gape calculations. The final column (comparing all species at a mandibular length of 60 cm) was used to directly compare the influence gape mechanics has on optimum prey depth. Table is updated from Young et al. (2012b), with our description of NHMUK PV R3939, PETMG:R176, PETMG: R60 and CAMSM J64267.

Species	Optimum gape angle	Optimum prey depth (as % of mandibular length)	Maximum known mandible length	Maximum known optimum prey depth	Optimum prey depth with a 60 cm long mandible
*Metriorhynchus superciliosus* NHMUK PV R3016	11	8%	88 cm	7.04 cm	4.8 cm
‘*Metriorhynchus*’ *brachyrhynchus* NHMUK PV R3804	10	7%	80.9 cm	5.66 cm	4.2 cm
*Tyrannoneustes* *lythrodectikos* GLAHM V972, NHMUK PV R3939, PETMG:R176, PETMG: R60, CAMSM J64267	15	13%	∼102 cm	13.26 cm	7.8 cm
*Geosaurus giganteus* NHMUK PV OR37020	16	13%	52 cm	6.76 cm	7.8 cm
*Dakosaurus maximus* SMNS 82043	19	15%	87.5 cm	13.13 cm	9 cm
*Dakosaurus andiniensis* Gasparini, Pol & Spalletti (2006)	23	19%	80 cm	15.2 cm	11.4 cm
*Plesiosuchus manselii* NHMUK PV R1089, NHMUK PV OR40103	24	21%	132.2 cm	27.76 cm	12.6 cm

In an evolutionary context, *Tyrannoneustes lythrodectikos* has a unique combination of plesiomorphic and derived characteristics related to wide-gape macrophagy (Young et al., 2013a). Plesiomorphically, it has: a moderately long tooth count (>17 alveoli per maxilla), a moderately long mandibular symphysis (10–12 pairs of dentary alveoli adjacent to the symphysis), and very poorly developed denticles that are non-contiguous along the carinae. However, it also has the following derived characteristics: increased tooth crown apicobasal length, an increased optimal gape, a shearing occlusion mechanism and enlarged supratemporal fenestrae. Therefore, there are three characteristics seen in *Tyrannoneustes* + Geosaurini that are possibly linked to wider gape feeding: (1) increased tooth length, (2) shearing occlusion mechanics, and (3) enlarged temporal musculature. The combination of increasing tooth crown apicobasal length and sophisticated occlusal patterns would have increased the potential shearing surface area (Young et al., 2012a; Young et al., 2013a). These adaptations are seen in extant marine predators, such as false killer whales and the great barracuda, and minimise the energy required to break large prey items into pieces small enough to consume (Grubich, Rice & Westneat, 2008; Young et al., 2012a). Increasing muscle force magnitude by enlarging the muscle cross-sectional areas is one way to compensate for low bite forces at wide gape (Bourke et al., 2008; Young et al., 2013a). Previously, outside Geosaurini only *Neptunidraco ammoniticus* was known to increase the supratemporal fenestrae diameter (Cau & Fanti, 2011; Young et al., 2013a), but it is now seen in *Tyrannoneustes lythrodectikos* (NHMUK PV R3939 and PETMG:R176). Dependent upon the evolutionary relationships of *Neptunidraco*, this characteristic could be either homoplastic within Geosaurinae, or alternatively, unites *Neptunidraco* with the subclade *Tyrannoneustes* + Geosaurini. Nevertheless, the wide-gape feeding adaptations seen in *Tyrannoneustes* + Geosaurini can be characterised as either: reducing the force required to break large prey items into smaller pieces, and/or possibly increasing the temporal muscle force magnitude.

Some members of Geosaurini have other adaptations linked to wide-gape macrophagy that are not present in *Tyrannoneustes lythrodectikos.* These include: shortening and broadening of the snout (resulting in the brevirostrine condition), deepening and shortening of the mandibular symphyses, low overall tooth count (<16 alveoli per maxilla and dentary), and ziphodont teeth which had contiguous denticles along the carinae (Pol & Gasparini, 2009; Andrade et al., 2010; Young et al., 2010; Young et al., 2012a; Young et al., 2012b; Young et al., 2013a; Young et al., 2013b). It has been hypothesised that the denticle morphology seen in *Tyrannoneustes* did not act as a ‘functional saw’, due to their heterogenic nature (i.e., non-contiguous along the carinae), being poorly defined, and lack of influence on carinal keel height (Young et al., 2013a). Moreover, true functional ziphodonty (well developed denticles that are contiguous along the carinae) is only known to occur within Geosaurini (Andrade et al., 2010; Young et al., 2013a). Serrated carinae are known to reduce the energy required to break prey items into pieces small enough to swallow, because these serrations facilitate slicing and cutting (Frazzetta, 1988; Purslow, 1991; Abler, 1992; Freeman & Weins, 1997; Evans & Sanson, 1998).

As the transmission of muscle force to bite force (mechanical advantage) is relatively lower at wide-gapes than at narrow-gapes (Dumont & Herrel, 2003), then adaptations that increase mechanical advantage would compensate for this. The foreshortening of the snout (i.e., reducing the out-lever)—and its resultant reduction in tooth count in Geosaurini—is the most obvious adaptation for increasing mechanical advantage. Interestingly, this may have evolved independently in each of the four Geosaurini genera, and in the metriorhynchine *Cricosaurus saltillensis* (Buchy, Young & Andrade, 2013). The early Kimmeridgian taxon *Torvoneustes coryphaeus* and other basal Geosaurini had a tooth count comparable to *Tyrannoneustes lythrodectikos* (Young et al., 2013a). Whereas the late Kimmeridgian-early Tithonian taxa *Torvoneustes carpenteri*, *Geosaurus giganteus*, *Dakosaurus maximus, D. andiniensis* and *Plesiosuchus manselii* all had a low (14 or fewer) maxillary tooth count (Fraas, 1902; Wilkinson, Young & Benton, 2008; Pol & Gasparini, 2009; Young & Andrade, 2009; Young et al., 2012b). If correct, then increasing mechanical advantage through snout foreshortening could be the last adaptation to wide-gape macrophagy that evolved in Geosaurinae.

## Conclusions

Here we describe the first known skulls of *Tyrannoneustes lythrodectikos* (PETMG:R176 and NHMUK PV R3939), and two lower jaws, one (PETMG:R60) possibly belonging together with PETMG:R176 and one (CAMSM J64267) from an individual smaller than the holotype. These specimens further highlight the mosaic nature of wide-gape macrophagous evolution in Geosaurinae, as like the mandible of the holotype, they have a combination of plesiomorphic and derived characteristics. Moreover, the skulls also have apomorphic characteristics, such as the elongate premaxillary posterior processes and the deep notches on the lateral surfaces of the maxillae for the dentary teeth and the involvement of the premaxilla in the first maxillary alveolus.

Based on our current understanding, *Tyrannoneustes lythrodectikos* was not the only macrophagous metriorhynchid in the Oxford Clay Formation. Young et al. (2013a) described a very large tooth crown which has well-developed denticles that were contiguous along both the mesial and distal carinae. These characteristics are only known within Geosaurini. It therefore appears that there was another macrophagous metriorhynchid in the Oxford Clay Sea, perhaps a member of Geosaurini. We hope that the careful description of museum specimens, and new discoveries, will further elucidate this tantalising possibility. Moreover, we plan to study these specimens in detail, investigating the feeding ecology, evolution and niche partitioning among these numerous sympatric taxa.

**Institutional Abbreviations**
BRSMG: Bristol City Museum & Art Gallery, Bristol, United Kingdom
BSPG: Bayerische Staatssammlung für Paläontologie und Historische Geologie, München, Germany
CAMSM: Sedgwick Museum of Earth Sciences, University of Cambridge, United Kingdom
GLAHM: Hunterian Museum, Glasgow, Scotland, United Kingdom
MOZ: Museo Provincial de Ciencias Naturales “Prof. Dr. Juan A. Olsacher”, Zapala, Neuquén Province, Argentina
NHMUK: Natural History Museum, London, England, United Kingdom
PETMG: Peterborough Museum, Peterborough, United Kingdom
SEC: Steve Etches Collection, Kimmeridge, United Kingdom
SMNS: Staatliches Museum für Naturkunde, Stuttgart, Germany

**Anatomical Abbreviations**
an: angular
ar: articular
bo: basioccipital
co: coronoid
cp: coronoid process
den: dentary
eo: exoccipital
fm: foramen magnum
for: foramina
fr: frontal
ic: internal carotids
jug: jugal
ls: laterosphenoid
max: maxillary
na: nasal
no: ‘notches’
oc: occipital condyle
pal: palatine
pa: parietal
po: postorbital
pr: proötic pra? prearticular fragment?
pre: premaxillary
prf: prefrontal
qu: quadrate
rp: reception pits
san: surangular
sdg: surangulodentary groove
sq: squamosal
su: supraoccipital
sp: splenial
X-XI-XII: cranial nerves ten to twelve
